# Doxorubicin-polyglycerol-nanodiamond conjugate is a cytostatic agent that evades chemoresistance and reverses cancer-induced immunosuppression in triple-negative breast cancer

**DOI:** 10.1186/s12951-019-0541-8

**Published:** 2019-10-17

**Authors:** Shen-Jun Yuan, Yong-Hong Xu, Chao Wang, Hui-Chao An, Hua-Zhen Xu, Ke Li, Naoki Komatsu, Li Zhao, Xiao Chen

**Affiliations:** 1 Department of Pharmacology, School of Basic Medical Sciences, Wuhan University, Donghu Avenue No.185, Wuhan, 430072 China; 2 Hubei Provincial Key Laboratory of Developmentally Originated Disease, Wuhan, 43007 China; 3 Department of Ophthalmology, Institute of Ophthalmological Research, Renmin Hospital of Wuhan University, Wuhan, 430060 China; 4 Center for Lab Teaching, School of Basic Medicine, Wuhan University, Donghu Avenue No.185, Wuhan, 430072 China; 5 Graduate School of Human and Environmental Studies, Kyoto University, Sakyo-ku, Kyoto, 606-8501 Japan; 6 State Key Laboratory of Radiation Medicine and Protection, School of Radiation Medicine and Protection & School for Radiological and Interdisciplinary Sciences (RAD-X), Collaborative Innovation Center of Radiation Medicine of Jiangsu Higher Education Institutions, Soochow University, Suzhou, 215123 Jiangsu China

**Keywords:** Doxorubicin-polyglycerol-nanodiamond conjugate, Triple-negative breast cancer, Chemoresistance, Immunosuppression, Immunochemotherapy

## Abstract

**Background:**

Triple negative breast cancer (TNBC) has the poorest prognosis of all breast cancer subtypes and is one of the most fatal diseases for women. Combining cytotoxic chemotherapy with immunotherapy has shown great promise for TNBC treatment. However, chemotherapy often leads to the development of chemoresistance and severe systemic toxicity compromising the immune functions that are crucial to anti-TNBC immune therapy. Tumor-induced immunosuppression also poses a great hindrance to efficacious anti-TNBC immunotherapy. Nanomedicine holds great promise to overcome these hurdles.

**Results:**

Doxorubicin-polyglycerol-nanodiamond conjugate (Nano-DOX) was firstly found to be a cytostatic agent to the 4T1 cells and displayed a lower apparent therapeutic potency than DOX. However, the tumor-bearing animals, particularly some key immune cells thereof, showed good tolerance of Nano-DOX as opposed to the severe toxicity of DOX. Next, Nano-DOX did not induce significant upregulation of P-gp and IL-6, which were demonstrated to be key mediators of chemoresistance to DOX in the 4T1 cells. Then, Nano-DOX was shown to downregulate tumor-derived granulocyte-colony stimulating factor (G-CSF) and suppresses the induction and tissue filtration of myeloid-derived suppressor cells (MDSCs) that are the principal effectors of cancer-associated systemic immunosuppression. Nano-DOX also alleviated the phenotype of MDSCs induced by 4T1 cells. Finally, Nano-DOX induced the 4T1 cells to emit damage associated molecular patterns (DAMPs) that stimulated the tumor immune microenvironment through activating key immune effector cells involved in anti-tumor immunity, such as macrophages, dendritic cells and lymphocytes in the tumor tissue.

**Conclusions:**

Nano-DOX is a cytostatic agent with good host tolerance which is capable of evading chemoresistance and reversing cancer-induced immunosuppression both at the systemic level and in the tumor microenvironment in TNBC. Our work presents Nano-DOX as an interesting example that a chemotherapeutic agent in nano-form may possess distinct biochemical properties from its free form, which can be exploited to join chemotherapy with immunotherapy for better treatment of cancer.
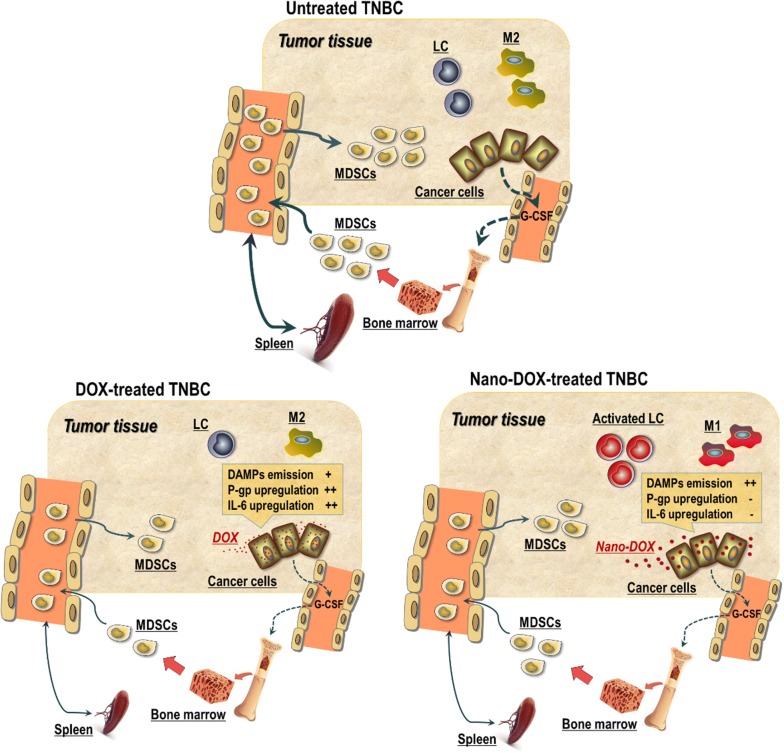

## Background

About 1 million women worldwide are diagnosed with breast cancer every year, among which 15–20% patients are estimated to be the triple-negative phenotype [1]. Triple-negative breast cancer (TNBC) carries a high risk of early recurrence and has a higher likelihood of visceral metastasis and poorer prognosis than other breast cancer subtypes [2]. Unlike other types of breast cancer, growth of TNBC cells are not fueled by estrogen, progesterone and epidermal growth factor since TNBC is negative for estrogen receptor (ER), progesterone receptor (PR), and overexpression of human epidermal growth factor receptor 2 (HER2) [3]. Hence, TNBC does not respond to hormone therapies or treatments that target these receptors. This leaves chemotherapy to be the primary systemic treatment for both early- and advanced-stage TNBC, which is currently applied as standard-of-care in the neoadjuvant (before surgery), adjuvant (after surgery), and metastatic settings [4]. Common chemotherapeutic drugs for TNBC treatment include anthracyclines, platinum drugs, taxanes, cyclophosphamide, 5-fluorouracil and etc. While TNBCs appear to be susceptible to chemotherapy initially, only a small portion (~ 20%) of patients can achieve sustained response and chemoresistance with multiple mechanisms rapidly develops in most patients leading to relapse of the disease [5]. Moreover, most chemotherapeutic drugs have systemic toxicity often causing severe collateral damages such as myelosuppression, immunosuppression, cardiotoxicity, neuropathy and myalgia. These therapeutic conundrums frequently lead to treatment failure wherefore TNBC has the worst overall outcome of all breast cancer subtypes and remains one of the deadliest diseases for women. It is thus of paramount importance to develop novel therapeutic approaches to TNBC treatment.

The emergence of immunotherapy, such as checkpoint inhibitors, tumor vaccines and adoptive cell therapy, has changed the landscape of cancer treatment and brought new hopes to TNBC patients [6]. Immunochemotherapy, a combination of immunotherapy and chemotherapy has been proposed as a novel promising strategy for TNBC treatment [7, 8]. While emerging results are encouraging about the efficacy of this strategy, certain obstacles still remain that hold off unleashing of its full therapeutic potential. As mentioned above, chemotherapy often inflicts severe toxicity on various immune cells that are crucial to anti-cancer immunity. More importantly, tumor-induced immunosuppression poses a bottleneck for efficacious anti-cancer immunotherapy. Tumor-induced immunosuppression refers to cancer cells harnessing the immune system in such a way that not only disables anti-cancer immunity but also facilitates tumor genesis, survival and progression. This process features coordinated mobilization of major immune regulatory components such as myeloid-derived suppressor cells (MDSCs), suppressive macrophages (Mφ), regulatory dendritic cells (DC) and T lymphocytes. Of global importance among these cells are MDSCs (known as Gr-1^+^/CD11b^+^ cells in mice), a heterogeneous population of immature myeloid cells with immunoregulatory potency to suppress lymphocyte-mediated innate and adaptive immunity [9]. Cancer cells release hematopoietic growth factors, particularly G-CSF and GM-CSF to stimulate the production of immature myeloid cells which are hijacked by the cancer thus giving rise to the MDSCs. MDSCs infiltrate peripheral lymphoid tissues (lymph nodes and spleen) and organs such as the liver and lungs as well as the tumor tissue, thus acting both systemically and in the local tumor environment. In the tumor microenvironment, MDSCs can be differentiated into suppressive DC, Mφ and granulocytes that further help to sustain local immunesupresssion. Chemoresistance, systemic toxicity and tumor induced-immunosuppression represent a difficult gap in realizing the potential of immunochemotherapy.

Numerous nano-based chemotherapeutic delivery systems have been devised mostly for the purpose of targeted tumor destruction but little attention has been paid to their immune activities though immune effects are implicated in the anti-tumor efficacy of certain chemotherapeutic agents [10]. We had also fabricated polyglycerol-functionalized nanodiamonds carrying doxorubicin (Nano-DOX) for tumor-targeted delivery of doxorubicin (DOX) [11]. Subsequent studies on glioblastoma models discovered some unique properties of Nano-DOX other than targeted tumor toxicity, e.g. good tolerance by immune cells and stimulation of the immunogenicity of cancer cells through inducing immunogenic cell death (ICD), which properties were then utilized to modulate the immune microenvironment of glioblastoma [12, 13]. As DOX is a first-line anti-TNBC chemotherapy, the present work was carried out initially to compare Nano-DOX and DOX for their therapeutic effects on mouse TNBC models. But some unexpected discoveries that we have made prompted us later to evaluate Nano-DOX from a more comprehensive perspective. By findings presented in this manuscript, we demonstrate Nano-DOX as an interesting example that nano-based chemo-drug devices may also serve to bridge the gap between chemotherapy and immunotherapy for the treatment of TNBC.

## Methods

### Materials

**Fig. 1:**
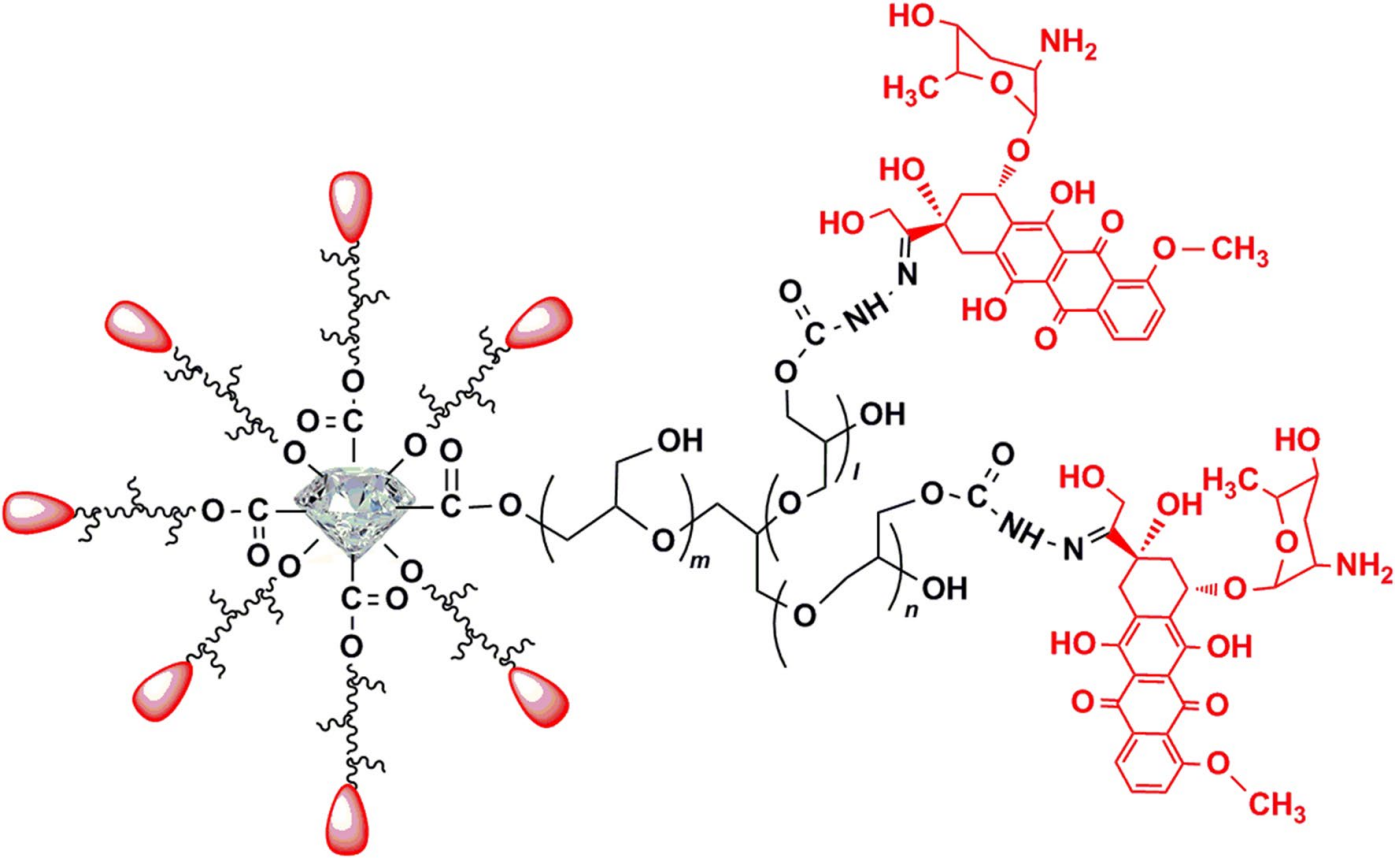
Composition and structure of Nano-DOX

This composite was synthesized on the basis of nanodiamonds (Nd, 4–5 nm in diameter) with a surface coating of polyglycerol (Nd-PG). DOX was loaded to the Nd-PG giving Nano-DOX. All functional moieties were covalently immobilized on the surface of Nd. Briefly, PG was grafted on Nd through ring-opening polymerization of glycidol via hydroxyl groups on the surface as starting points. To load DOX on the Nd-PG, partial hydroxyl groups on Nd-PG were converted to hydrazine groups (Nd-PG-NH-NH_2_) through organic reactions. Next, DOX was conjugated with Nd-PG-NH-NH_2_ through hydrazone bonding, which can be cleaved at a weak acidic pH. Nano-DOX has an aqueous hydrodynamic diameter of 83.9 ± 32.3 nm and good solubility in physiological solutions. Zeta potential of Nano-DOX dispersed in water was 23.8 ± 7.2 mV. Synthesis and characterization of Nano-DOX were described in detail in a previously published paper [11]. Figure 1 shows the structural composition of Nano-DOX. Nano-DOX stock solution in water was kept at 4 °C and was sonicated in a water bath for 3 min before being diluted with culture medium into working concentrations. All concentrations and dosages of Nano-DOX in the experiments were normalized to DOX.

### Cell models

4T1 mouse mammary carcinoma cell line was purchased from the Cell Bank of Shanghai Institutes for Biological Sciences (Shanghai, China). Mouse bone marrow-derived DC (BMDC), mouse spleen-derived lymphocytes and mouse bone marrow-derived Mφ (BMDM) were prepared according to previously published protocols [12, 13]. MDSCs were prepared from the spleen of 4T1 tumor-bearing mice according to a published protocol [14]. All cells were cultured in RPMI-1640 media (HyClone), supplemented with 10% fetal bovine serum (FBS, Sigma-Aldrich), 100 U/mL penicillin, and 100 µg/mL streptomycin (all from Gibco-Invitrogen) at 37 °C, in a 5% CO_2_ humidified incubator.

## 4T1 TNBC mouse model, drug treatment and fluorescent imaging

Female 6- to 8-week-old wild-type BALB/c mice were purchased from Hubei Provincial Center for Disease Control and Prevention (Wuhan, China) and bred in our animal facility under specific-pathogen-free conditions with fresh water and rodent diet available at all times. All animal procedures were carried out under protocols that complied with the Institutional Animal Care and Use Committee Guidelines for Ethical Conduct in the Care and Use of Animals. To reproduce mammary tumors, approximately 5 × 10^5^ live 4T1 cells suspended in 100 µL of PBS (Servicebio, China) were inoculated subcutaneously into the fourth abdominal mammary fat-pad of the mice. The appearance of tumors was monitored daily by palpating the inoculation area with gloved fingers, which happened approximately between day 5 and day 7 post inoculation, with tumors becoming palpable in all animals by day 7. When a tumor was palpable, the animals were randomly divided into 5 groups (8 mice per group). Animals in the test groups were then treated with injections of Nano-DOX or DOX (2 and 5 mg/kg body weight) via the tail vein at 2-day intervals for 3 weeks [15]. Meanwhile, control animals were given intravenous injections of normal saline. The animals were monitored for their body weight on a daily basis. At 24 h after the last administration, in vivo fluorescent images were acquired with a Bruker Xtreme BI imaging system (BRUKER, USA). Representative photographs showing the distribution of drug fluorescence in 4T1 xenograft-bearing mice were presented in Additional file 1: Fig. S1. The animals were then sacrificed and blood, tissues and organs of interest were harvested and fixed in 4% paraformaldehyde for hematoxylin and eosin (HE) staining, immumohistochemical (IHC) and immunofluorescence (IF) analysis or were processed immediately for cell analysis by FACS. Excised tumor xenografts were also imaged for detection of Nano-DOX or DOX fluorescence before paraformaldehyde fixation. Representative photographs showing tumor drug distribution were presented in Additional file 1: Fig. S2. Particularly, the mammary tumors and the spleens were weighed and photographed and serum G-CSF levels were quantified using ELISA kits (Multisciences, EK2692/2).

### Assay of cell viability, proliferation and apoptosis

Optimally growing 4T1 tumor cells in 96-well plates with a density of 7 × 10^3^ cells/well were treated with Nano-DOX and DOX at concentrations up to 4 μg/mL in culture medium, 100 μL per well for 24 h. BMDC, lymphocytes, BMDM and MDSCs each at a density of 2 × 10^4^ cells/100 µL/well in 96-well plates were treated with Nano-DOX or DOX (0.5–4 μg/mL) for 24 h. Cell viability was assayed with a CCK-8 kit as instructed in the manual provided by the manufacturer (Dalian Meilun Biotechnology Co., LTD., China). For cell proliferation assay, CFDA-SE (Sigma-Aldrich)-labeled 4T1 were treated with 2 μg/mL of Nano-DOX or DOX for 24 h. The cells were then taken out at 0 h and 24 for FACS analysis of CFSE staining, the decay of which is proportional to the rate of cell proliferation [12]. For cell apoptosis assay, 4T1 cells were seeded in 24-well plates (NEST Biotechnology, Wuxi, China) with a seeding density of 2 × 10^5^ cells/well and treated with 2 μg/mL of Nano-DOX or DOX (0.5 mL/per well) for 24 h. Cells were then harvested and apoptosis were determined by staining cells with Annexin V-FITC (or Annexin V-APC) (Multisciences, China) and FACS according to the manufacturer’s protocol. In brief, 2 × 10^5^ cells were re-suspended in 100 µL binding buffer and 5 µL Annexin V was added to each sample which was then incubated for 15 min at room temperature in the dark. An additional 400 µL binding buffer was then added to the reaction prior to FACS analysis.

### Assay of MDR-1 expression, P-gp transport activity and effect of P-gp blockage on cell apoptosis

4T1 cells were harvested after treatment of Nd-PG, Nano-DOX or DOX at 2 μg/mL for 24 h. Expression of MDR-1 mRNA and protein (i.e. P-gp) were then detected by RT-PCR, western blotting, immunofluorescent staining, confocal microscopy and FACS. To verify P-gp transport activity in 4T1 cells, the cells in two parallel wells in a 24-well plate were first loaded with 100 nM of rhodamine-123 (R123, Sigma), a specific P-gp substrate, for 30 min. Cells in one well were then lifted and analyzed by FACS for cellular R123 fluorescence. The cells in the other well were left in fresh culture medium with or without 10 μM of verapamil (VRP, Macklin), a specific P-gp blocker, for 3 h and then taken out for FACS analysis of cellular R123 fluorescence. The same procedure was also repeated on DOX and Nano-DOX to determine if they are substrates of P-gp efflux activity in 4T1 cells. Alternatively, 4T1 cells were incubated with DOX or Nano-DOX for 3 h in the presence or absence of VRP and cellular uptake of DOX or Nano-DOX was determined by FACS. To determine the effect of P-gp blockage on cell apoptosis, 4T1 cells were incubated with 2 μg/mL of Nano-DOX or DOX in the presence or absence of VRP for 24 h. The cells were then lifted, stained for annexin v immunofluorescence and then analyzed by FACS.

### Analysis of IL-6 expression and IL-6’s role in cell apoptosis

4T1 cells in 6-well plates with a seeding density of 6 × 10^5^ cells/well were treated with 2 μg/mL of Nano-DOX or DOX for 24 h. IL-6 mRNA levels in the samples were determined by RT-PCR. IL-6 levels in the culture medium supernatants were determined using an ELISA kit (4A Biotech Co., Ltd.) according to the manufacturer’s instructions. To establish IL-6’s protective role against apoptosis in 4T1 cells, IL-6 expression was silenced by small interference RNA (siRNA). For silencing IL-6, IL-6-directed siRNA (molecular weight (MW): 13,266.1, SenseSeq: GGACTGATGCTGGTGACAA) and control siRNA (molecular weight (MW): 13,296.2, SenseSeq: UUCUCCGAACGUGUCACGUTT) were purchased from Wuhan GeneCreate Biological Engineering Co., Ltd. After the cells were grown up to 40 ~ 60% confluence in Opti-MEM, the appropriate siRNAs were transfected into cells using Lipofectamine™ 3000 reagent (Invitrogen, CA, USA) according to the manufacturer’s instructions. RT-PCR was conducted after 24-h transfection to evaluate the silencing efficacy of the siRNA on target gene expression. To assess the validity of our siRNA experiment, we tested three types of control including transfection control, untreated control, and negative control in all siRNA experiments. IL-6-silenced 4T1 cells in 24-well plates with a seeding density of 2 × 10^5^ cells/well were treated with 2 μg/mL of Nano-DOX or DOX in the presence or absence of mouse recombinant IL-6 (50 ng/mL, PeproTech) for 24 h before being analyzed for FACS analysis of annexin v immunofluorescent staining.

### Phenotyping analysis of MDSCs

At the end of 3-week treatment of Nano-DOX or DOX, BM and blood cells were extracted from 4T1 tumor-bearing mice. BM cells were collected from mouse femurs and tibias, then washed twice with PBS. After depleting erythrocytes, purified BM cells were collected for FACS analysis. Peripheral blood samples were collected into 1.5-mL tubes containing 3.8% sodium citrate anticoagulant. After lysis of red blood cells (RBCs), the cells were washed three times with ice-cold PBS containing 1% of BSA (Biosharp, China) for FACS analysis. Alternatively, bone marrow cells from normal mice were prepared and treated with the 4T1 conditioned culture medium for 72 h. The cells were then collected for FACS analysis. We confirmed the viability of the cells to be greater than 90% with trypan blue staining. To analyze the phenotypic change of MDSCs after Nano-DOX or DOX treatment by FACS, MDSCs were gated according to immunofluorescent staining of Gr-1 and CD11b.

### Assay of DAMPs emission

4T1 in 24-well plates with a seeding density of 1 × 10^5^ cells/well were treated with Nano-DOX or DOX at 2 μg/mL for 24 h. Cell surface HSP90 and CRT were then detected by immunofluorescent staining, FACS and confocal microscopy. Culture medium supernatants were collected and HMGB1 levels were determined with ELISA kit (Elabscience, China) according to the manufacturer’s instructions and ATP levels determined with a Chemiluminescence ATP Determination Kit (Beyotime, S0027, China) and a luminometer (Tecan, Spark 10 M).

### Phenotyping analysis of Mφ

4T1 (5 × 10^5^ cells/well) were treated with Nano-DOX (2 μg/mL) for 24 h in 6-well plates and then the conditioned culture medium (ND-CM) were collected. M2 in 24-well plates (2 × 10^5^ cells/well) were treated with ND-CM or 2 μg/mL of Nano-DOX for 24 h. The cells were then harvested for (1) analysis of cell surface markers CD86 by immunofluorescent staining and FACS, (2) Western blotting analysis of GBP5 expression, and (3) extraction of total mRNA for RT-PCR analysis of gene expression of iNOS, CD206 and TGF-β.

### Assay of DC activation and DC-driven activation of lymphocytes

DC in 24-well plates (2 × 10^5^ cells/well) were treated with ND-CM or 2 μg/mL of Nano-DOX for 24 h. The cells were then taken and measured by FACS for surface expression of CD40, CD80, CD83 and MHC-II (surface markers indicating DC activation). In lymphocyte activation experiments, the ND-CM or 2 μg/mL of Nano-DOX were used to treat DC (2 × 10^5^ cells/well) for 24 h. Lymphocytes with or without CFDA-SE labelling were then added in each well at a density of 1 × 10^6^ cells/well and the cell cultures were maintained for another 24 h before being harvested for analysis. Proliferation of the lymphocytes were evaluated by FACS measurement of the decay of CFSE fluorescence. Surface expression of CD69 (maker of lymphocytes activation) in the CD4^+^ and CD8^+^ T lymphocytes were analyzed by immunofluorescent staining and FACS.

### Immunofluorescent staining

For fluorescent immunostaining in the 4T1 cells, cells were fixed with paraformaldehyde (4%) in 1× PBS and then blocked with 5% BSA in 1× PBS at 37 °C for 1 h. Cells were then incubated with primary Ab against MDR-1 (SantaCruz, sc-55510), HSP90 (Abcam, ab13495), or CRT (Abcam, ab92516) at 4 °C overnight. The stained cells were washed 3 times with PBST (1% Tween-20 in 1× PBS), incubated with Alexa Flour 488-conjugated secondary antibody (Proteintech, SA00006-2) at 37 °C for 2 h further stained, washed 3 times with 1× PBS. Finally, cell were stained with Hoechst 33342 (5 μg/mL) for 15 min at room temperature and washed 3 times with 1× PBS. Cells were then examined under a confocal microscope (Leica-LCS-SP8-STED, Germany). For fluorescent immunostaining in the mice tissue samples, paraffin sections were incubated with the following primary antibodies: rabbit anti-mouse CD11b (Proteintech, 20991-1-AP) and rat anti-mouse Gr-1 (eBioscience, 14-5931-82) at 4 °C for overnight. The sections were washed with Tris-buffer saline and subsequently stained with the following secondary antibodies: CY3-labelled goat anti-rabbit, FITC-labelled goat anti-rat (Life technologies), with the nuclei being counterstained with 4′,6-diamidino-2-phenylindole (DAPI). Samples were imaged using an Olympus confocal microscope.

### Flow cytometry analysis

Cellular fluorescence was acquired on a Beckman Cytoflex flow cytometer (CA, USA). Antibodies for flow cytometry analysis included Pacific Blue-conjugated antibodies to Gr-1 (BioLegend, 108430), APC-conjugated antibodies to CD11b (BioLegend, 101212), eFluor 450-conjugated antibodies to CD40 (eBioscience, 48-0402-80), APC-conjugated antibodies to CD80 (BioLegend, 104713), APC-conjugated antibodies to CD83 (BioLegend, 121509), APC-conjugated antibodies to CD86 (eBioscience, 17-0862-81), eFluor 450-conjugated antibodies to MHC-II (BioLegend, 107620), APC-conjugated antibodies to CD4 (BioLegend, 100516), eFluor 450-conjugated antibodies to CD8a (eBioscience, 48-0081-82), FITC-conjugated antibodies to CD69 (eBioscience, 11-0691-82). DOX and Nano-DOX fluorescence was acquired in the PE channel. Cells were stained with antibodies for 30 min at 4 °C before they were subjected to FACS analysis. At least 1 × 10^4^ cells/per sample were acquired.

### Quantitative real-time PCR (qRT-PCR)

Total RNA from 4T1 tumor cells or Mφ was extracted by using TRIzol reagent (Invitrogen) and checked for purity and concentration with A260/A280 reading (Spark 10 M, Tecan). mRNA contained in 1000 ng of RNA was reverse transcribed into cDNA using a transcriptor cDNA synthesis kit (PrimeScript RT Master Mix, TaKaRa). cDNA samples were amplified in a CFX96 Real-time System (Bio-Rad Laboratories, Hercules, CA, USA) and SYBR Green Master Mix (TaKaRa). The primers used were below: MDR-1 sense: 5′-GCTGGTTTGATGTGCACGATGTTGG-3′; antisense: 5′-ATTTTGTCACCAATTCCTTCATTAA-3′; IL-6 sense: 5′-CGGAGAGGAGACTTCACAGAG-3′; antisense: 5′-ATTTCCACGATTTCCCAGAG-3′; Bcl-2 sense: 5′-CTGGCATCTTCTCCTTCCAG-3′; antisense: 5′-GACGGTAGCGACGAGAGAAG-3′; G-CSF sense: 5′-CATGGCTCAACTTTCTGCCCA-3′; antisense: 5′-TAGGTGGCACACAACTGCTC-3′; iNOS sense: 5′-TGGAGCGAGTTGTGGATTGTC-3′; antisense: 5′-GTGAGGGCTTGGCTGAGTGA -3′; CD206 sense: 5′-AGGGAAACAATACCTTGAACCCAT-3′; antisense: 5′-GAGCTGGGAGAAGATGAAGTCAA-3′; TGF-β sense: 5′-TACAGGGCTTTCGCTTCAGTG-3′; antisense: 5′-GTGGAGCTGAAGCAGTAGTTGG-3′; GM-CSF sense: 5′-TCCGGAAACGGACTGTGAAACA-3′; antisense: 5′-TGCCACATCTCTTGGTCCCTTT-3′; IL-8 sense: 5′-TTTCCACCGGCAATGAAG-3′; antisense: 5′-TAGAGGTCTCCCGAATTGGA-3′; β-actin sense: 5′-TGAGAGGGAAATCGTGCGTGAC-3′; antisense: 5′-GCTCGTTGCCAATAGTGATGACC-3′. Fold changes in mRNA expression between treatments and controls were determined by the △CT method. The data were normalized to a β-actin reference.

### Western blotting

Cells subjected to required treatments in six-well plates were rinsed twice with ice-cold PBS and lysed in RIPA buffer with 1% protease inhibitor cocktail. Cell lysates were cleared by centrifugation and protein concentration was determined with a bicinchoninic acid (BCA) assay kit (Sigma). Equal protein aliquots (25 μg) were fractionated by SDS-PAGE and transferred to a PVDF membrane. The membranes were blocked with 5% fat-free milk in TBST and incubated with antibodies against MDR-1 (SantaCruz, sc-55510), GBP5 (Proteintech, 13220-1-AP) and GADPH overnight at 4 °C. Protein bands were imaged using a horseradish peroxidase-conjugated secondary antibody and ECL and the films were exposed with a Bio Imaging system (Syngene).

### IHC analysis

Antibodies for immunohistochemical analysis included rabbit anti-Ki67 (Abcam, ab15580), mouse anti-PCNA (BOSTER, BM0104), rabbit anti-Caspase-3 (Proteintech, 19677-1-AP), rabbit anti-CD3 (Abcam, ab5690), rabbit anti-F4/80 (Abcam, Ab100790), mouse anti-MDR-1 (SantaCruz, sc-55510), rabbit anti-IL-6 (Bioss, bs-0782R), Rabbit anti-G-CSF (Bioss, bs-1023R), rabbit anti-HSP90 (Abcam, ab13495), rabbit anti-CRT (Abcam, ab92516), rabbit anti-HMGB1 (Abcam, ab79823), rabbit anti-CD80 (Bioss, bs-2211R), mouse anti-CD86 (Abcam, ab213044), rabbit anti-GBP5 (Proteintech, 13220-1-AP), rabbit anti-MHC-II (Bioss, bs-4298R), rabbit anti-CD206 (Abcam, ab64693), rabbit anti-CD4 (Bioss, bs-0766R), rabbit anti-CD8 (Bioss, bs-0648R), rabbit anti-CD69 (MULTI SCIENCES, ab2304), rabbit anti-foxp3 (Bioss, bs-10211R). Paraffin sections (5 μm) were dewaxed and rehydrated, antigen repaired with sodium citrate for 20 min, then incubated in 3% hydrogen peroxide for 10 min at room temperature. The paraffin sections were blocked with 5% BSA for 30 min, stained with antibodies overnight at 4 °C, washed with PBS and stained with secondary antibody (PV-9000, ZSGB-BIO) for 1 h at room temperature. Diaminobenzidine (DAB, ZLI-9018, ZSGB-BIO) was applied for coloration for 5 min at room temperature. Hematoxylin was used to stain the nucleus.

### Data analysis

All data are presented as mean ± SD and analyzed by using the GraphPad software. Comparison between two groups for statistical significance were performed with unpaired Student’s t test. For more groups, one-way ANOVA followed by Neuman–Keuls post hoc test was used.

## Results

### Nano-DOX displayed lower apparent anti-TNBC potency but higher host tolerance than DOX

**Fig. 2:**
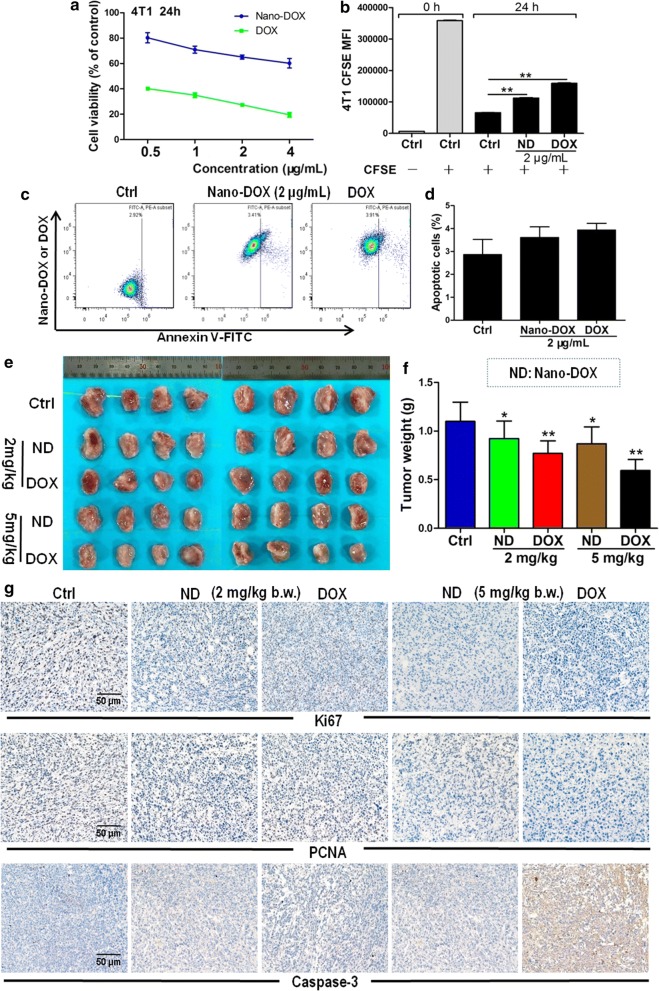
Anti-TNBC efficacy of Nano-DOX in comparison with DOX. **a** Effects of Nano-DOX and DOX on the viability of 4T1 cells in vitro assayed by the CCK-8 test. **b** Effects of Nano-DOX and DOX on the proliferation of 4T1 cells in vitro assayed by CFSE staining. **c**, **d** Apoptosis of 4T1 cells after 24-h treatment of Nano-DOX or DOX assayed by annexin V immunofluorescent staining and FACS. **e**, **f** Size and weight of orthotopic 4T1 tumor xenografts in mice at the end of 3-week treatment of Nano-DOX or DOX. **g** Immunohistochemical staining of Ki67, PCNA (markers of cancer cell proliferation), and caspase 3 (marker of cancer cell apoptosis) in mouse orthotopic 4T1 tumor xenografts at the end of 3-week treatment of Nano-DOX or DOX. (Duration of Nano-DOX or DOX treatment was 24 h for the in vitro cell experiments.) In FACS analysis, geometric means were used to quantify fluorescence intensity. Values were mean ± SD (n = 3 for in vitro experiments and n = 8 for in vivo experiments, *p < 0.05, **p < 0.01)

We first looked at the anti-TNBC efficacy and host toxicity of Nano-DOX in comparison with DOX. In-vitro experiments showed that Nano-DOX (0.5-4 μg/mL) suppressed the viability and proliferation of 4T1 cells but with a lower potency than DOX (Fig. 2a, b). Nano-DOX (2 μg/mL) caused little apoptosis in the 4T1 cell (Fig. 2c, d), which was not unexpected as previous studies had also showed and explained Nano-DOX’s inability to elicit apoptosis in glioblastoma cells [16]. It came as a surprise that nor did DOX (2 μg/mL) cause much apoptosis of 4T1 cells (Fig. 2c, d), as DOX is a well-known apoptosis inducer and 2 μg/mL of DOX was adequate to induce significant apoptosis in other cancer models we had studied [16]. Mechanisms for this observation are elucidated in subsequent studies. Therapeutic efficacy of Nano-DOX and DOX on mice bearing orthotopic breast cancer xenografts was consistent with in vitro results. Tumors that received Nano-DOX were of bigger size and weight than those treated with DOX of the same dosages (2 and 5 mg/kg body weight) at the end of the treatment duration (Fig. 2e, f). Nano-DOX also suppressed the expression of Ki67 and PCNA, markers of tumor cell proliferation and growth, to a lesser degree than DOX. See Fig. 2g for immunohistological staining of Ki67 and PCNA in 4T1 xenografts. In keeping with the in vitro apoptosis analysis, tumors treated with Nano-DOX either at 2 or 5 mg/kg body weight showed little sign of apoptosis as indicated by the immunohistological staining of caspase-3 while DOX caused remarkable extent of apoptosis at 5 mg/kg body weight (Fig. 2g). These results indicate that Nano-DOX has a lower apparent anti-TNBC potency than DOX, which is proliferation inhibition of the TNBC cells rather than cell killing.

**Fig. 3:**
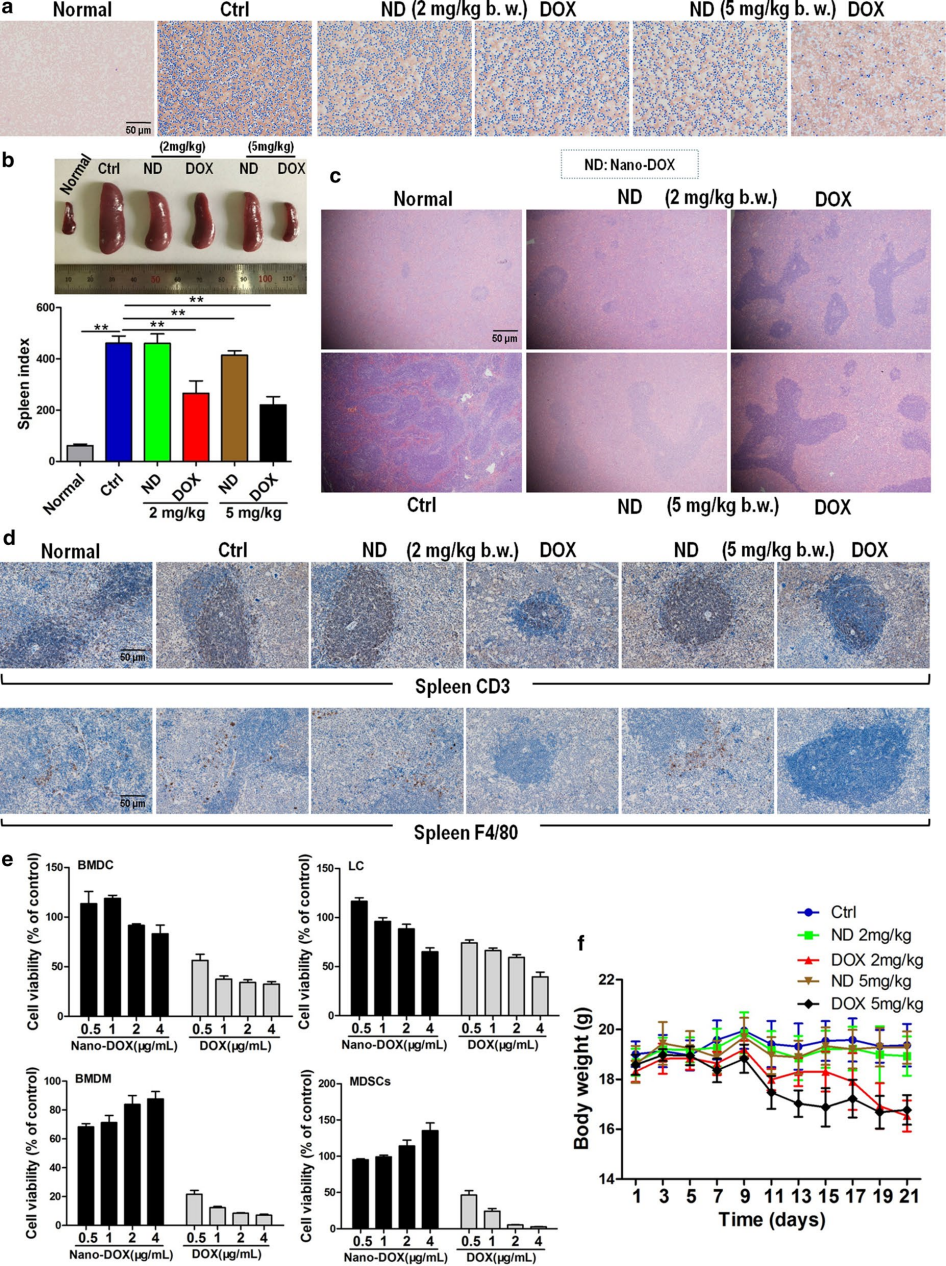
Anti-TNBC efficacy and host toxicity of Nano-DOX in comparison with DOX. **a** Peripheral blood smears from 4T1-tumor bearing mice at the end of 3-week treatment of Nano-DOX or DOX. **b** Spleen size and spleen index of 4T1-tumor bearing mice at the end of 3-week treatment of Nano-DOX or DOX. **c** H&E staining of spleen tissues from 4T1-tumor bearing mice at the end of 3-week treatment of Nano-DOX or DOX. **d** Immunohistochemical staining of CD3 (marker of lymphocytes) and F4/80 (marker of macrophages) in spleen tissues from 4T1-tumor bearing mice at the end of 3-week treatment of Nano-DOX or DOX. **e** Effects of Nano-DOX and DOX on the viability of bone marrow-derived dendritic cells (BMDC), lymphocytes (LC), bone marrow-derived macrophages (BMDM) and myeloid-derived suppressor cells (MDSCs) assayed by CCK-8 test. **f** Body weight (b.w.) changes of 4T1-tumor bearing mice at the end of 3-week treatment of Nano-DOX or DOX. Duration of Nano-DOX or DOX treatment was 24 h for the ex vivo cell experiments. Values were mean ± SD (n = 5 for in vitro experiments and n = 8 for in vivo experiments, *p < 0.05, **p < 0.01)

One unique feature of the 4T1 TNBC model is high constitutive secretion of granulocyte-colony stimulating factors (G-CSF), a glycoprotein that stimulates the bone marrow to produce large amount of leukocytes, mostly granulocytes, which go into the peripheral blood and then infiltrate tissues such as the spleen, lymph nodes, liver, lungs, as well as the tumor. Particularly, the spleen becomes ostentatiously enlarged due to massive granulocyte infiltration, a condition called splenomegaly [17]. As shown in Fig. 3a, b, both DOX and Nano-DOX alleviated the tumor-induced granulocytosis and splenomegaly. The spleen tissues also showed downsized red pulp in DOX- and Nano-DOX-treated tumor-bearing mice indicating reduced granulocyte infiltration (Fig. 3c). These observations are further evidence of Nano-DOX’s anti-TNBC action which, though, is of a lesser intensity than that of DOX.

DOX also reduced lymphocyte and macrophage infiltration in the spleen as indicated by the diminished staining of CD3 and F4/80 (markers of lymphocytes and Mφ, respectively) in the spleen tissues (Fig. 3d). This suggests that DOX’s direct toxicity on leukocytes may have also contributed to the alleviation of the granulocytosis and splenomegaly in tumor-bearing mice. In support thereof, mouse bone marrow-derived dendritic cell (BMDC), lymphocytes, bone marrow-derived macrophage (BMDM) and MDSCs were found invariably very sensitive to DOX’s toxicity (Fig. 3e). By contrast, Nano-DOX did not significantly reduce lymphocyte and macrophage infiltration in the spleen and the in vitro mouse leukocytes maintained their viability in the presence of Nano-DOX except for the BMDC and lymphocytes at the highest concentration of 4 μg/mL (Fig. 3d, e). In line with these observations, tumor-bearing mice receiving DOX (2 and 5 mg/kg body weight) displayed a progressive decline in body weight until the end of the treatment indicating significant systemic toxicity whereas the Nano-DOX-treated animals maintained their body weight similar to the control group evidencing good tolerability (Fig. 3f). These findings speak strongly for a much lesser host toxicity of Nano-DOX than DOX.

### Nano-DOX, as opposed to DOX, did not induce chemoresistance mediated by P-glycoprotein and IL-6 in TNBC cells

**Fig. 4:**
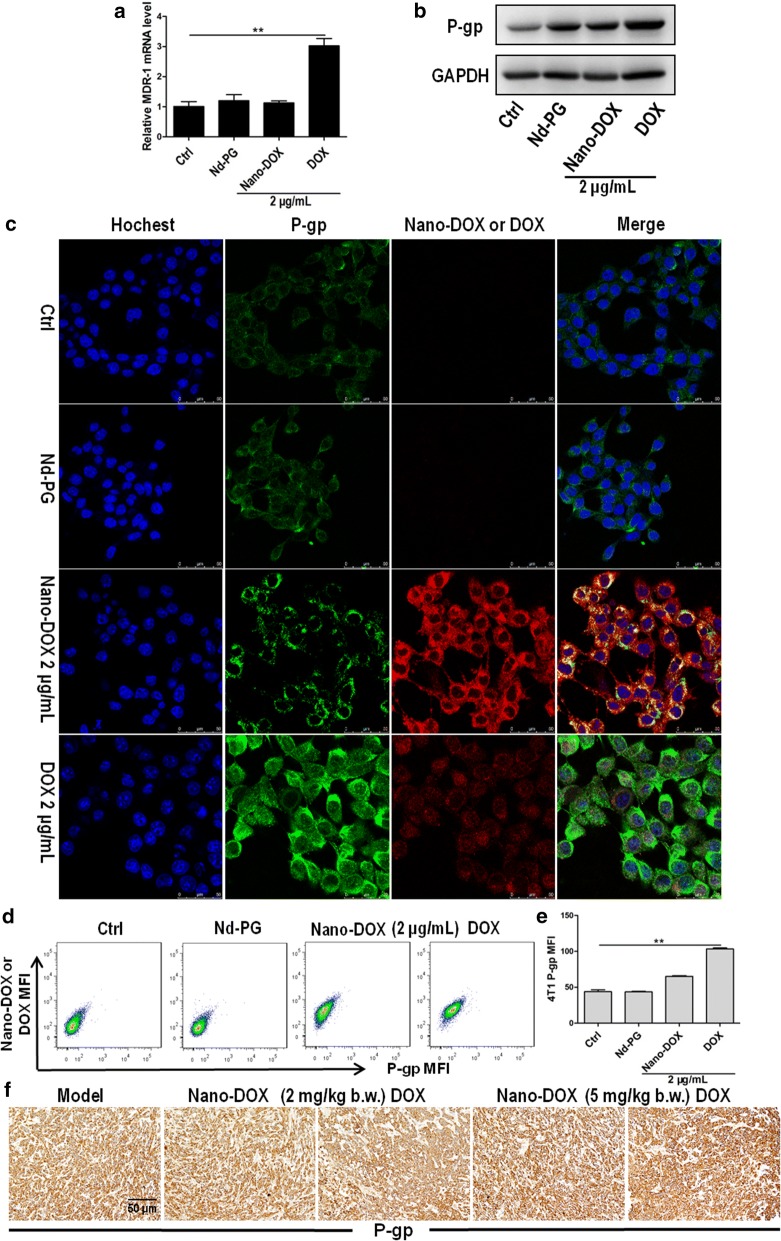
Effects of Nano-DOX and DOX on the expression of P-gp (MDR-1 protein) in 4T1 cells. **a** RT-PCR analysis of MDR-1 mRNA levels in in vitro 4T1 cells treated with polyglycerol-functionalized nanodiaminds (Nd-PG), Nano-DOX and DOX. **b** Western blotting analysis of P-gp expression in in vitro 4T1 cells treated with polyglycerol-functionalized nanodiaminds (Nd-PG), Nano-DOX and DOX. **c** Confocal microscopy of P-gp immunofluorescent staining in in vitro 4T1 cells treated with polyglycerol-functionalized nanodiaminds (Nd-PG), Nano-DOX and DOX. Blue fluorescence was nuclear staining by Hoechst 33342; green fluorescence was P-gp staining and red fluorescence came from Nano-DOX or DOX. **d**, **e** FACS analysis of P-gp immunofluorescent staining in in vitro 4T1 cells treated with polyglycerol-functionalized nanodiaminds (Nd-PG), Nano-DOX and DOX. **f** Immunohistochemical staining of P-gp in mouse orthotopic 4T1 tumor xenografts at the end of 3-week treatment of Nano-DOX or DOX. Duration of treatment was 24 h for the in vitro cell experiments. In FACS analysis, geometric means were used to quantify fluorescence intensity. Values were mean ± SD (n = 3, *p < 0.05, **p < 0.01)

**Fig. 5:**
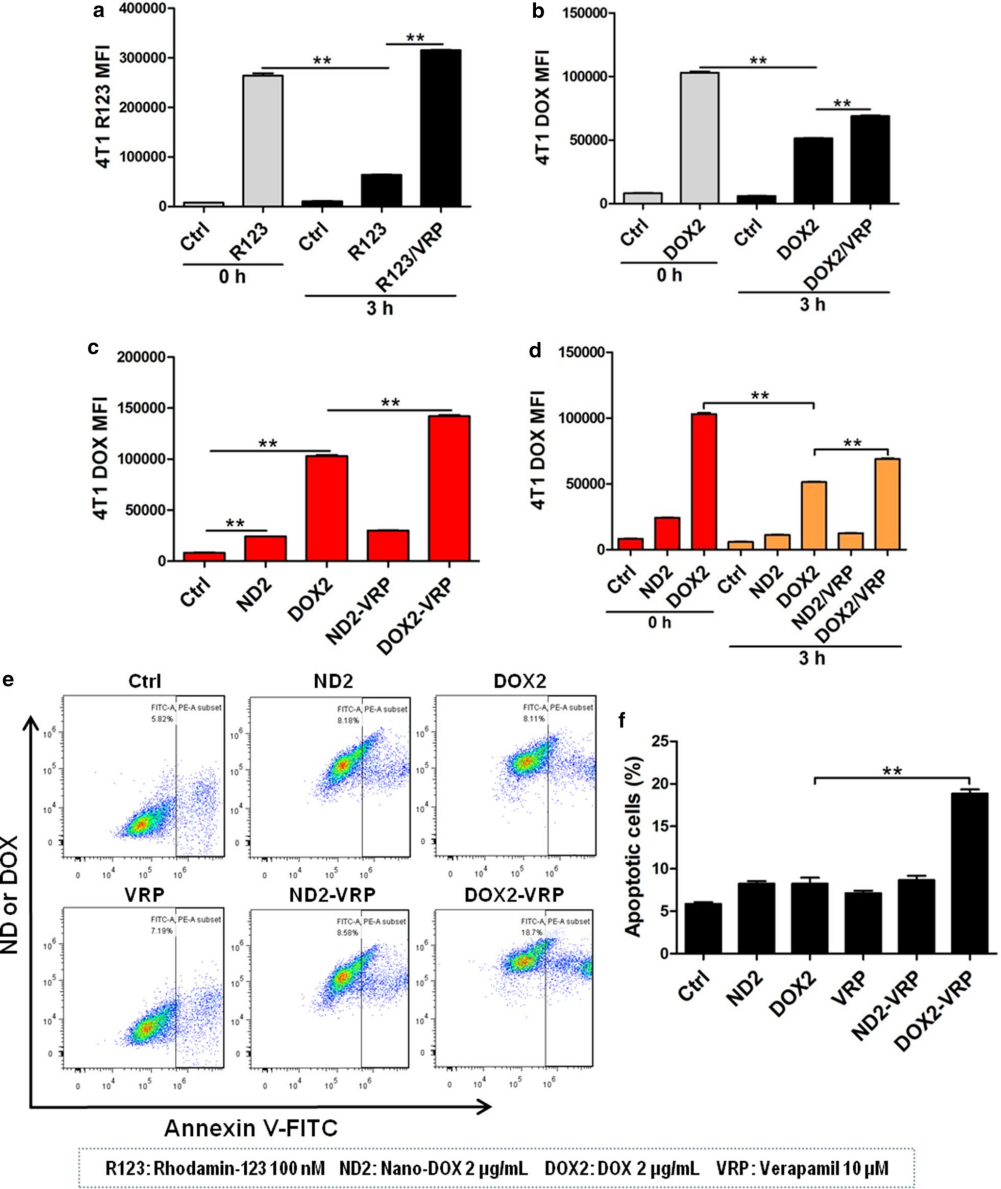
Verification of P-gp transport activity in 4T1 cells and the effect of P-gp blockage on cellular uptake, efflux and toxicity of Nano-DOX and DOX. **a** Efflux of rhodamine-123 (R123), a specific P-gp substrate, from 4T1 cells and the effect of verapamil (VRP), a specific P-gp inhibitor, on R123 efflux. **b** Efflux of DOX from 4T1 cells and VRP’s effect on DOX efflux. **c** VRP’s effect on the uptake of Nano-DOX and DOX by 4T1 cells. **d** VRP’s effect on the efflux of Nano-DOX and DOX from 4T1 cells. **e**, **f** VRP’s effect on apoptosis in 4T1 cells treated with Nano-DOX or DOX (All experiments in this section were performed in vitro and data were acquired through FACS.) In FACS analysis, geometric means were used to quantify fluorescence intensity. Values were mean ± SD (n = 3, *p < 0.05, **p < 0.01)

As the 4T1 cells did not exhibit much apoptosis when treated with 2 μg/mL of DOX which was otherwise an effective cytocidal agent to previously studied cancer models, we suspected that the 4T1 cells must possess certain resistance mechanisms that protect against DOX. P-glycoprotein (P-gp) quickly came to mind upon considering cancer resistance to DOX. P-gp is a membrane-bound transport protein belonging to the ATP-binding cassette (ABC) transporter family that actively translocate their substrates across the cell membrane against the concentration gradient using energy derived from ATP hydrolysis [18]. Over-expressions of P-gp reduces uptake and increases efflux of multiple drugs thus preventing the drugs from reaching therapeutic concentrations at their target sites within a cell, leading to multidrug resistance (MDR). DOX is a well-known substrate of P-gp and has been reported to induce P-gp in multiple cancers [19]. Indeed, in our work, we not only detected constitutive expression of P-gp in the 4T1 cells but also observed massive P-gp up-regulation induced by DOX both in vitro and in vivo (Fig. 4). In stark contrast, Nano-DOX only up-regulated P-gp to an unremarkable extent (Fig. 4). These observations led to the speculation that (1) P-gp-mediated efflux may be a key contributor to the 4T1 cells’ resistance to DOX but not Nano-DOX, and (2) blocking P-gp transport activity would increase intracellular retention of DOX thus augmenting its cytotoxic effects but would not affect Nano-DOX’s effect. For verification, we first confirmed DOX as a substrate of P-gp transport in the 4T1 cells. As shown in Fig. 5a, b, verapamil (VRP), a competitive inhibitor of P-gp, blocked the efflux both of DOX and R123, a specific P-gp transport substrate. Next, we demonstrated that P-gp blockage by VRP both increased DOX uptake and decreased its efflux in the 4T1 cells whereas neither the uptake nor efflux of Nano-DOX was much affected by VRP, indicating that Nano-DOX was not subject to the transport activity of P-gp (Fig. 5c, d). Consistently, VRP was further found to markedly enhance 4T1 cell apoptosis induced by DOX but not Nano-DOX (Fig. 5e, f). Taken together, these discoveries adequately prove that P-gp has little impact on Nano-DOX’s action on the 4T1 cells and Nano-DOX does not induce P-gp, upregulation of which is a key mechanism of DOX resistance in 4T1 cells.

**Fig. 6:**
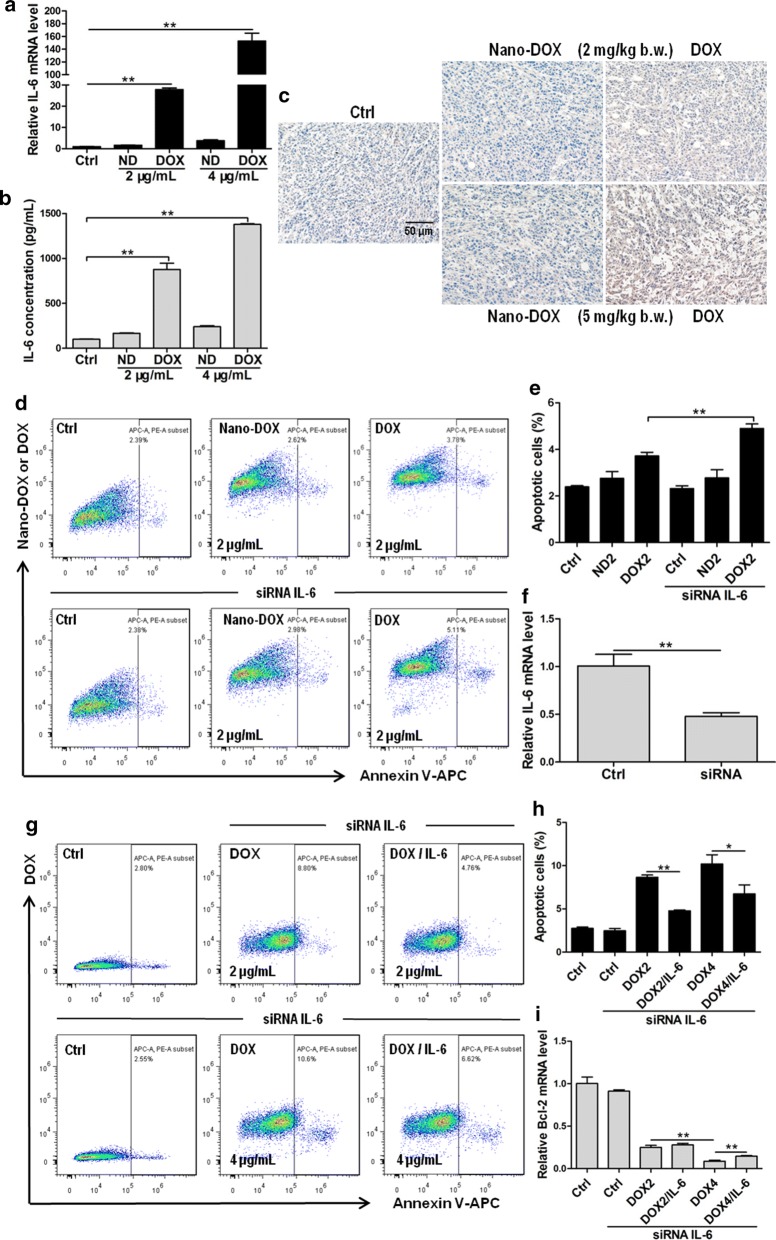
Effects of Nano-DOX and DOX on the expression of IL-6 in 4T1 cells and the effect of IL-6 on apoptosis in 4T1 cells treated with Nano-DOX or DOX. **a** RT-PCR assay of IL-6 mRNA levels in in vitro 4T1 cells treated with Nano-DOX or DOX. **b** ELISA analysis of IL-6 secretion from in vitro 4T1 cells treated with Nano-DOX or DOX. **c** Immunohistochemical staining of IL-6 in 4T1 tumor xenografts at the end of 3-week treatment of Nano-DOX or DOX. **d, e** Effect of IL-6 silence on apoptosis in in vitro 4T1 cells treated with Nano-DOX or DOX, assayed by immunostaining and FACS. **f** Verification of IL-6 knock-down at the mRNA level, assayed by RT-PCR. **g**, **h** Rescue effect of exogenous IL-6 on DOX-induced apoptosis in in vitro 4T1 cells with IL-6 knock-down, assayed by immunostaining and FACS. **I:** Rescue effect of exogenous IL-6 on DOX-induced Bcl-2 mRNA downregulation in in vitro 4T1 cells with IL-6 knock-down, assayed by RT-PCR. Duration of Nano-DOX or DOX treatment was 24 h for the in vitro cell experiments. In FACS analysis, geometric means were used to quantify fluorescence intensity. Values were mean ± SD (n = 3, *p < 0.05, **p < 0.01)

Steep upregulation of IL-6 by DOX was another unexpected finding in our work (Fig. 6a–c). IL-6 is a versatile pro-cancer cytokine known to promote cancer survival, growth, invasion, and metastasis through multiple mechanisms [20]. Particularly, autocrine IL-6 has been reported to enhance the anti-apoptosis capacity of cancer cells, which is a key mechanism of resistance to multiple therapies [21, 22]. This also appeared to be the case in our study as silence of IL-6 increased DOX-induced 4T1 cell apoptosis as indicated by the annexin v staining and Bcl-2 expression, which could be rescued by exogenous IL-6 (Fig. 6d–i). By contrast, IL-6 knockdown hardly increased apoptosis in Nano-DOX-treated 4T1 (Fig. 6d, e). These observations indicate that Nano-DOX does not induce IL-6-mediated resistance to DOX.

### Nano-DOX mitigated TNBC’s induction of MDSCs with different potency than DOX

**Fig. 7:**
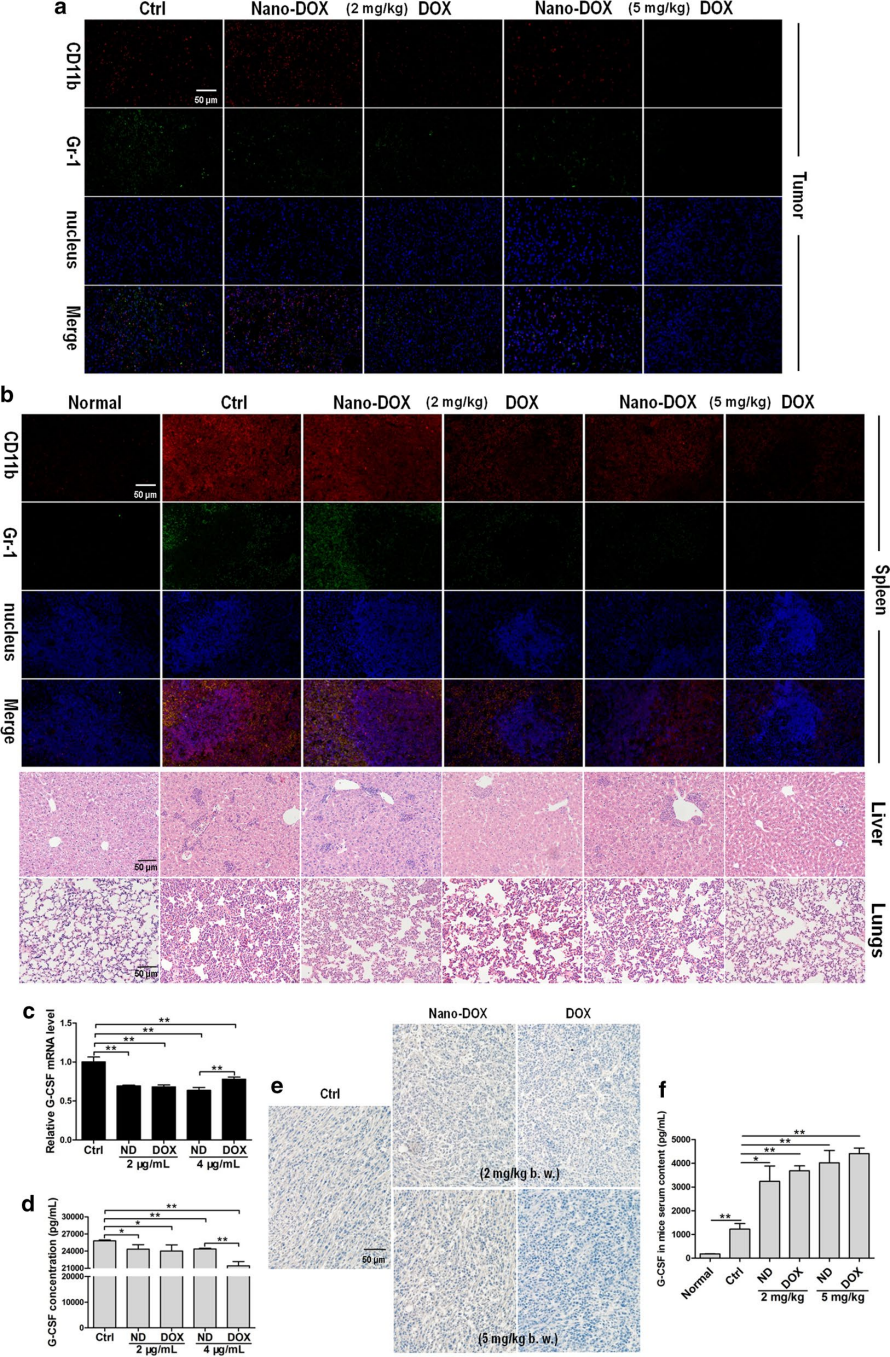
Effects of Nano-DOX and DOX on MDSCs infiltration in tissues and G-CSF expression. **a** Immunofluorescent staining of MDSCs markers CD11b and Gr-1 in 4T1 tumor xenografts at the end of 3-week treatment of Nano-DOX or DOX. **b** Immunofluorescent staining of MDSCs markers CD11b and Gr-1 in spleen and H&E staining of granulocytes in liver and lungs. **c** RT-PCR analysis of G-CSF mRNA levels in in vitro 4T1 cells treated with Nano-DOX or DOX. **d** ELISA analysis of G-CSF secretion from in vitro 4T1 cells treated with Nano-DOX or DOX. **e** Immunohistochemical staining of G-CSF in 4T1 tumor xenografts at the end of 3-week treatment of Nano-DOX or DOX. **f** Serum levels of G-CSF in 4T1 tumor-bearing mice at the end of 3-week treatment of Nano-DOX or DOX, assayed by ELISA. (Duration of Nano-DOX or DOX treatment was 24 h for the in vitro cell experiments.) Values were mean ± SD (n = 3 for in vitro experiments and n = 5 for in vivo experiments, *p < 0.05, **p < 0.01)

**Fig. 8:**
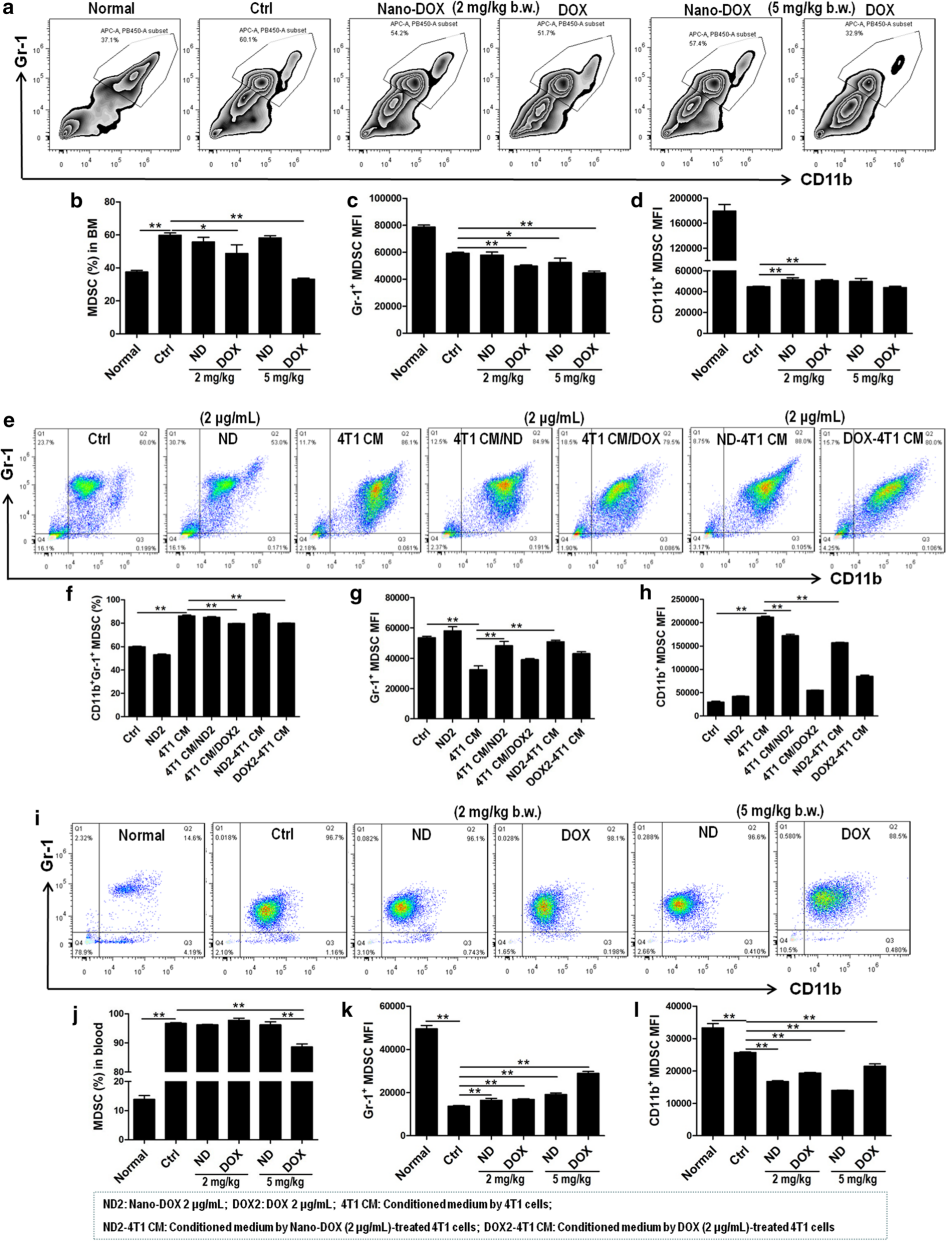
Effects of Nano-DOX and DOX on the phenotype of MDSCs. **a**–**d** Proportion of MDSCs (Gr-1^+^/CD11b^+^) in bone marrow and expression of MDSCs markers (Gr-1 and CD11b) in bone marrow MDSCs from 4T1 tumor-bearing mice at the end of 3-week treatment of Nano-DOX or DOX. **e**–**h** Proportion of MDSCs (Gr-1^+^/CD11b^+^) in ex vivo bone marrow and expression of MDSCs markers (Gr-1 and CD11b) in ex vivo bone marrow. **i**–**l** Proportion of MDSCs (Gr-1^+^/CD11b^+^) in the blood and expression of MDSCs markers (Gr-1 and CD11b) in blood MDSCs from 4T1 tumor-bearing mice at the end of 3-week treatment of Nano-DOX or DOX. (Duration of Nano-DOX or DOX treatment was 72 h for all ex vivo cell experiments and cells were analyzed by immunofluorescent staining and FACS.) In FACS analysis, geometric means were used to quantify fluorescence intensity. Values were mean ± SD (n = 3 for ex vivo experiments and n = 4 for in vivo experiments, *p < 0.05, **p < 0.01)

As already described above, a substantial leukocytosis is a characteristic of the 4T1 TNBC model (Fig. 3a). Further phenotyping analysis showed the circulating leukocytes were predominantly MDSCs (> 96%) (Fig. 8i, j) and extensive MDSCs infiltration were detected in the tissues of tumor, spleen, liver and lungs (Fig. 7a, b), indicating a systemic presence of MDSCs. Since both DOX and Nano-DOX alleviated the tumor-induced granulocytosis and splenomegaly (Fig. 3a, b), decreased MDSCs infiltration were expected and indeed observed in the tumor, spleen, liver and lungs (Fig. 7a, b). As tumor-derived G-CSF has been established as the principal driver of leukocytosis associated with many types of cancer [23], we posited that DOX and Nano-DOX might have downregulated G-CFS expression in 4T1 cells thus accounting for the abated granulocytosis (Fig. 3a) and tissue MDSCs infiltration (Fig. 7a, b). Indeed, both DOX and Nano-DOX were found to decrease G-CSF expression in 4T1 cells both in vitro and in vivo (Fig. 7c–e). To our surprise, however, serum level of G-CSF in the tumor-bearing mice did not decline but instead saw a remarkable lift in response to either DOX or Nano-DOX (Fig. 7f). This paradoxical rise in serum G-CSF level is frequently reported in clinical cancer patients and ascribed to a compensatory reaction to chemotherapy’s myelosuppression activity [24, 25]. In support of this observation, bone marrow MDSCs expansion in 4T1 tumor-bearing mice was found suppressed by DOX, and Nano-DOX with a much lesser severity (Fig. 8a, b), which is also in line with the extenuated granulocytosis shown in Fig. 3a. The bone marrow MDSCs in vivo also displayed a weak phenotype shift in response to Nano-DOX or DOX, i.e. a slight downregulation of Gr-1 and upregulation of CD11b (Fig. 8a, c, d). Interestingly, neither Nano-DOX nor DOX significantly suppressed expansion of ex vivo MDSCs directly induced by 4T1 cell-conditioned culture medium (Fig. 8e, f). However, Nano-DOX and DOX reversed the phenotype of ex vivo bone marrow MDSCs induced by 4T1 cells. As shown in Fig. 8e, g, h, 4T1 cell-conditioned culture medium induced a sharp downregulation of Gr-1 and upregulation of CD11b. Both Nano-DOX and DOX markedly alleviated this phenotype change of MDSCs, only with different potency (Fig. 8e, g, h). Consistently, a similar alleviation of tumor-induced phenotype was also detected in vivo in circulating MDSCs in response to DOX and Nano-DOX. As shown in Fig. 8i–k, besides a massive expansion, circulating MDSCs in 4T1 tumor-bearing mice were characterized by a much lower-than-normal expression of Gr-1 indicating an immature state as the expression level of Gr-1 directly correlates with granulocyte differentiation and maturation. In both DOX- and Nano-DOX-treated mice, circulating MDSCs displayed a significant upregulation of Gr-1 indicating a more differentiated phenotype (Fig. 8i, k). At the same time, MDSCs expression of CD11b, a surface protein important for cell adherence to endothelium, also decreased in response to DOX and Nano-DOX, which may reflect a lessened tissue-infiltrating capacity of the circulating MDSCs (Fig. 8i, l). Taken together, the above results are solid evidence that Nano-DOX could mitigate the induction of MDSCs by TNBC.

### Nano-DOX reversed the immunosuppressive microenvironment of TNBC

**Fig. 9:**
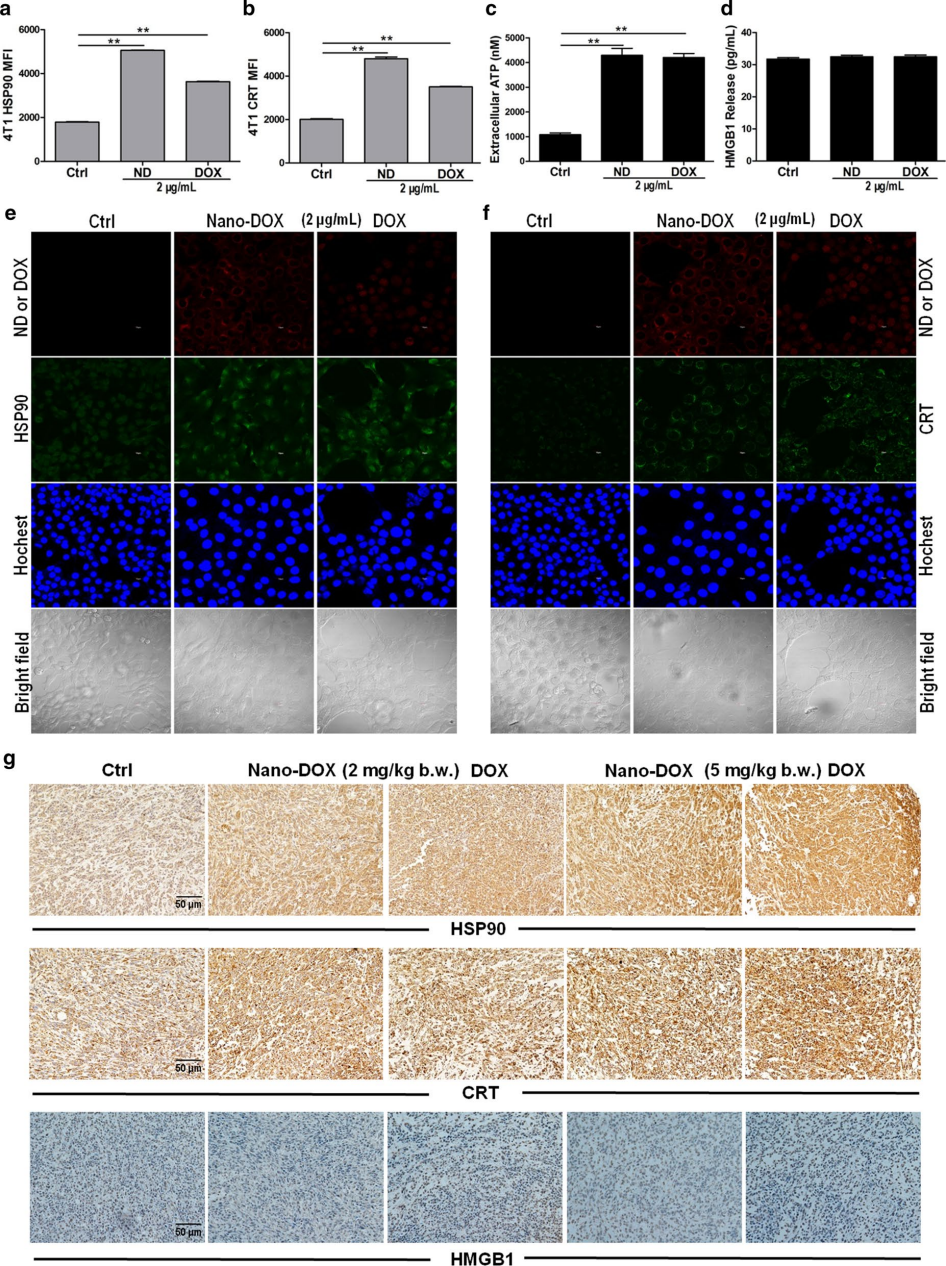
Effects of Nano-DOX and DOX on the emission of DAMPs in 4T1 cells. **a**, **b** FACS analysis of immunostaining of HSP90 and CRT in in vitro 4T1 cells treated with Nano-DOX or DOX. Representative FACS histograms are presented in Additional file 1: Fig. S5. **c** Bioilluminance assay of ATP released from in vitro 4T1 cells treated with Nano-DOX or DOX. **d** ELISA assay of HMGB1 released from in vitro 4T1 cells treated with Nano-DOX or DOX. **e** Confocal microscopy of HSP90 immunostaining in in vitro 4T1 cells treated with Nano-DOX or DOX. **f** Confocal microscopy of CRT immunostaining in in vitro 4T1 cells treated with Nano-DOX or DOX. **g** Immunohistochemical staining of HSP90, CRT and HMGB1 in 4T1 tumor xenografts at the end of 3-week treatment of Nano-DOX or DOX. Duration of Nano-DOX or DOX treatment was 24 h for the in vitro cell experiments. In FACS analysis, geometric means were used to quantify fluorescence intensity. Values were mean ± SD (n = 3, *p < 0.05, **p < 0.01)

**Fig. 10:**
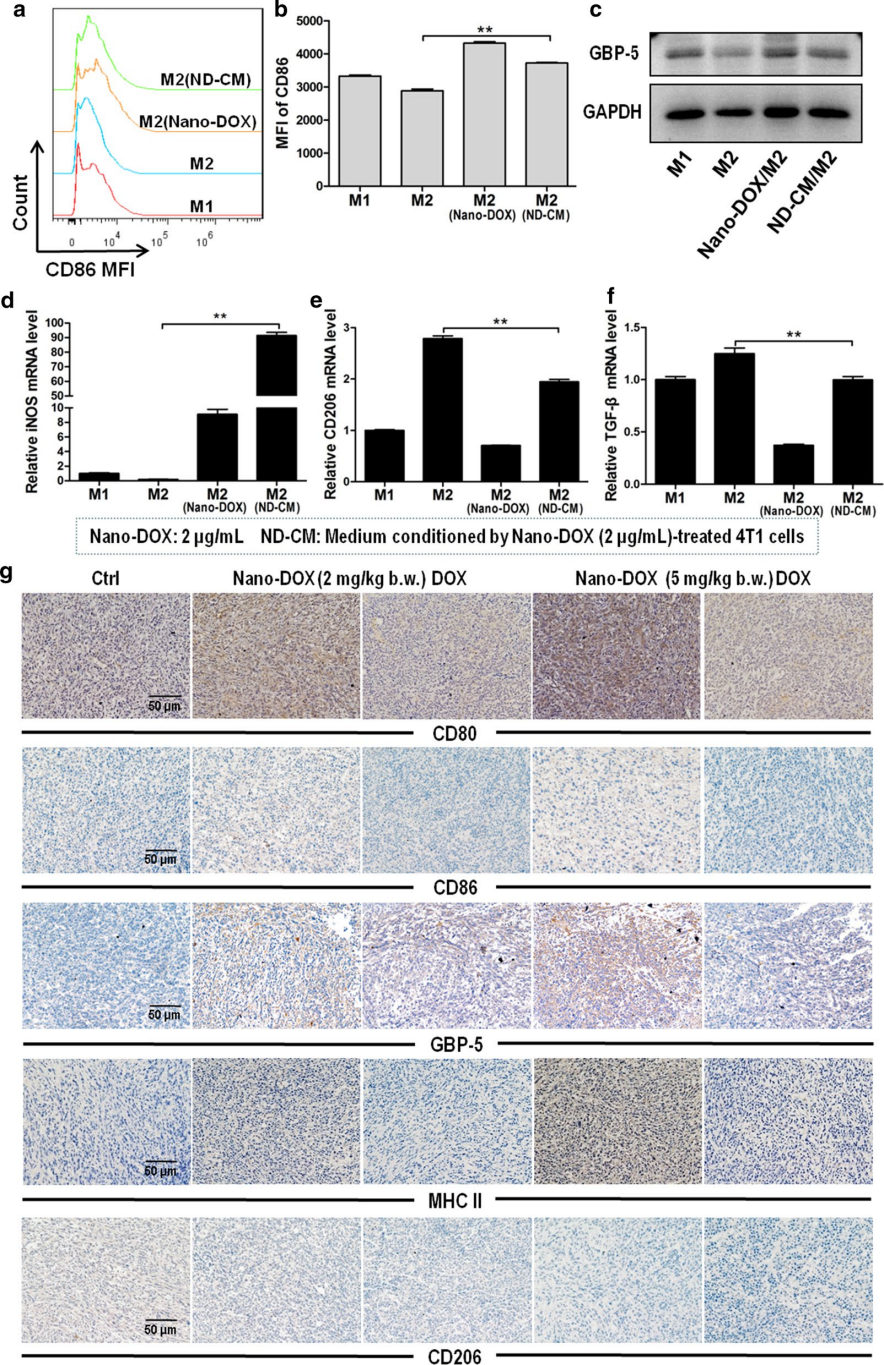
M2–M1 phenotypic shift of tumor-associated macrophages induced by Nano-DOX-treated 4T1 cells. **a**, **b** Immunostaining of CD86 (an M1 marker) in type-2 macrophages (M2) assayed by FACS. **c** Expression of GBP5 (an M1 marker) in M2 assayed by western blotting. **d** mRNA expression of iNOS (an M1 marker) in M2 assayed by RT-PCR. **e**, **f** mRNA expression of CD206 and TGF-β (M2 markers), respectively in M2 assayed by RT-PCR. **g** Immunohistochemical staining of CD80, CD86, GBP5, MHC-II (M1 markers), and CD206 (an M2 marker) in 4T1 tumor xenograts at the end of 3-week treatment of Nano-DOX or DOX. In the ex vivo cell experiments, type-1 and type-2 macrophages (M1 and M2) were activated from mouse bone marrow-derived macrophages according to published protocols. M2 were treated with Nano-DOX (2 μg/mL) or culture medium conditioned by Nano-DOX (2 μg/mL)-treated 4T1 cells (ND-CM). Duration of Nano-DOX or Nano-DOX-CM treatment was 24 h. In FACS analysis, geometric means were used to quantify fluorescence intensity. Values were mean ± SD (n = 3, *p < 0.05, **p < 0.01)

**Fig. 11:**
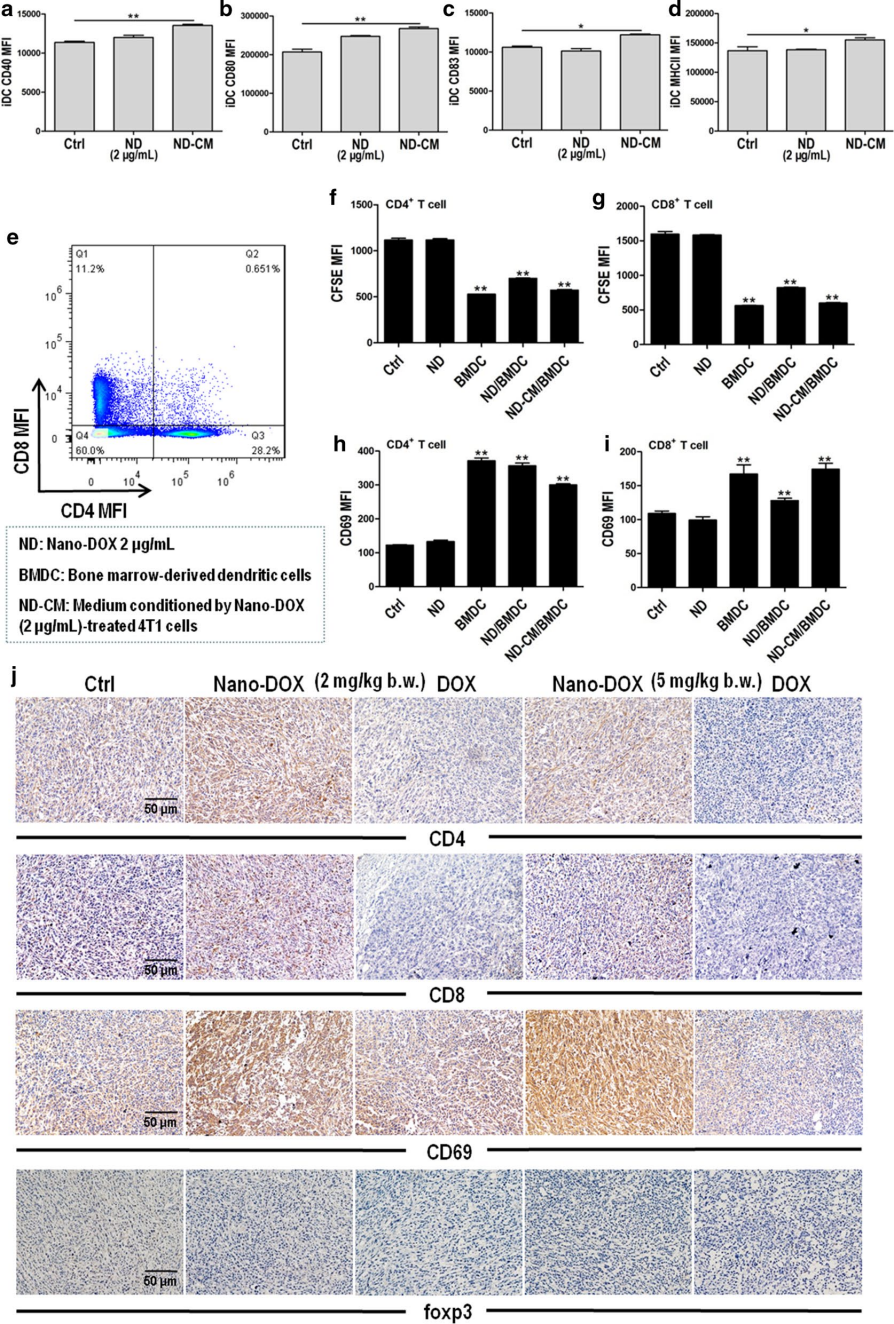
Activation of DC and T lymphocytes by Nano-DOX-treated 4T1 cells. **a**–**d** Immunostaining of CD40, CD80, CD83 and MHC-II (markers of DC activation) in bone marrow-derived DC (BMDC) assayed by FACS. Representative FACS histograms are presented in Additional file 1: Fig. S6. **e** Spleen-derived CD4^+^ and CD8^+^ T lymphocytes characterized by immunofluorescent staining and FACS.**f**, **g** Proliferation of CD4^+^ and CD8^+^ T lymphocytes, assayed by CFSE staining and FACS. Representative FACS histograms are presented in Additional file 1: Fig. S7. **h**, **i** Immunostaining of CD69 (a marker of T lymphocyte activation) in CD4^+^ and CD8^+^ T lymphocytes, assayed by FACS. Representative FACS dot plots are presented in Additional file 1: Fig. S8**. j** Immunohistochemical staining of CD4, CD8, CD69 and foxp3 (a marker of Treg) in 4T1 tumor xenografts at the end of 3-week treatment of Nano-DOX or DOX. In the ex vivo cell experiments, BMDC were first treated with Nano-DOX (2 μg/mL) or culture medium conditioned by Nano-DOX (2 μg/mL)-treated 4T1 cells (ND-CM) for 24 h. Spleen-derived lymphocytes were then co-cultured with the BMDC in the same system for another 24 h. In FACS analysis, geometric means were used to quantify fluorescence intensity. Values were mean ± SD (n = 3, *p < 0.05, **p < 0.01)

Besides the MDSCs, major immunosuppressive components in the tumor microenvironment (TME) include the TAMs, and regulatory T cells (Tregs). These suppressive cells, under the coordination of the cancer cells, inhibit the functions of CD4^+^ and CD8^+^ T cells infiltrating the tumor [26]. We have previously demonstrated Nano-DOX’s potency to stimulate the immunogenicity of glioblastoma cells [12, 13]. This capacity depends on Nano-DOX’s property to induce emission of antigens and damage associated molecular patterns (DAMPs) from the tumor cells. DAMPs are powerful endogenous adjuvants that could reactivate the anti-inflammatory type-2 TAMs (M2) into an immunostimulatory type-1 phenotype (M1). Moreover, tumor cell-derived DAMPs and antigens could cause enhanced DC activation which then drive the activation of CD4^+^ and CD8^+^ T cells infiltrating the tumor tissue thus subverting the immunosuppressive TME [27]. In the present study, although the 4T1 mouse mammary carcinoma cells are known to be poorly immunogenic, Nano-DOX was still found, both in vitro and in vivo, to elicit marked emission of three out of four typical DAMPs, i.e. HSP90, CRT and ATP, only leaving HMGB1 unchanged (Fig. 9). Consequently, the TAMs exhibited a M2–M1 phenotype shift in response to Nano-DOX-treated 4T1 cells both in vitro and in tumor xenografts. See Fig. 10 for the changes in markers of M1 activation (CD86, CD80, GBP-5, iNOS, MHC-II) and M2 activation (CD206, TGF-β). Moreover, Nano-DOX-treated 4T1 cells also induced in vitro DC activation and consequential lymphocyte activation. See Fig. 11a–i for the changes in action markers of DC and CD4^+^ and CD8^+^ T cells. These in vitro observations were substantiated by our in vivo study where Nano-DOX treated-4T1 tumor xenografts displayed markedly increased infiltration and activation of CD4^+^ and CD8^+^ T cells (marked by CD69) and diminished infiltration of suppressive Treg cells (marked by foxp3) (Fig. 11j). Notably, DOX’s effect on the tumor-infiltrating CD4^+^ and CD8^+^ T cells was of a similar pattern to Nano-DOX but with a lesser magnitude (Fig. 11j). In summary, these results affirm Nano-DOX’s capacity to reprogram the immunosuppressive microenvironment of TNBC.

## Discussion

Numerous nano-based delivery systems have been devised for targeted and efficient delivery of chemotherapeutic agents in malignant tumors. While nearly all cases have reported enhanced anti-cancer therapeutic efficacy and reduced collateral damage of non-malignant tissues at the same time, the question has been rarely asked whether a nano-formed drug acts to the same effects and by the same mechanisms as the original drug in free form. In our case, Nano-DOX was originally designed for cancer-targeted delivery of DOX and Nano-DOX indeed showed selective toxicity to cancer cells [11]. While DOX is a cytocidal agent used as first-line chemotherapy for many cancers, our previous work on glioblastoma cell models has demonstrated Nano-DOX to be essentially an inducer of autophagy rather than apoptosis [27]. In the present work using the 4T1 TNBC model, which was the first to evaluate Nano-DOX’s therapeutic anti-tumor efficacy, Nano-DOX consistently showed to be principally a cytostatic agent with a lesser therapeutic potency than free DOX (Fig. 2). Loss of DOX’s potency to kill at first glance seems to put Nano-DOX at a disadvantage compared with DOX. However, Nano-DOX does not lose its killing potency without gains. One big obstacle in cancer chemotherapy is its toxicity to non-malignant tissues and organ causing adverse effects that sometimes can be intolerable and DOX is a good case in point. As shown in the present work, DOX-treated animals displayed a continuous decrease in body weight indicating significant systemic toxicity whereas Nano-DOX-treated animals maintained stable body weight similar to control (Fig. 3f). Thus, reduced systemic toxicity is an apparent upside of Nano-DOX. Notably, immune cells such as DC, lymphocytes, Mφ and MDSCs that are otherwise very sensitive to DOX’s toxicity showed very good tolerance to Nano-DOX (Fig. 3e). We in previous studies have even successfully used some immune cells such as monocytes, Mφ and DC as carriers to actively deliver Nano-DOX in intracranial glioblastoma xenografts [12, 13, 28]. These observations, besides attesting to Nano-DOX’s low toxicity to non-malignant cells, have significant implications for anti-tumor immune therapy which depends on the viability and functions of these immune cells. Another point worth pointing out is that, although Nano-DOX displays a lower apparent potency than DOX at the tested doses, given Nano-DOX’s good host tolerability, the agent is expected to have an increased anti-tumor efficacy at higher doses at which DOX may have intolerable host toxicity. Moreover, Nano-DOX displayed selective tumor distribution over DOX (Additional file 1: Fig. S1), which is expected to be further enhanced by incorporation of a targeting moiety such as the cyclic ArgeGlyeAsp (RGD) peptide we have previously described [11].

Chemotherapy is the application of cellular poisons to kill the cancer cells by disrupting vital biochemical processes. The majority of the cancer cell population are initially sensitive to and wiped out by a given therapeutic agent but a few cells survive the poisonous stress through certain adaptation mechanisms such as decreased drug activation and increased drug inactivation, increased drug export and decreased intracellular drug accumulation, inhibition of apoptosis, and deregulated autophagy [29]. Expansion of these cells often gives rise to tumors that are not only resistant to the original agent but many other unrelated drugs, a phenomenon termed multi-drug resistance (MDR). Chemoresistance, especially MDR, is the principle cause of failure of chemotherapy. The mouse mammary carcinoma 4T1 cells in the present study showed remarkable resistance to DOX (Fig. 2c, e and Additional file 1: Fig. S3) and two mechanisms of resistance have been clearly identified i.e. induction of P-gp mediated drug export and upregulation of the pro-survival, anti-apoptotic interleukin 6 (IL-6) (Figs. 4, 5, 6). DOX-based combination chemotherapy is frequently required for TNBC treatment. However, the substrate spectrum of P-gp covers a variety of chemotherapeutic agents of different molecular structures and action mechanisms [30]. Autocrine IL-6 suppresses common apoptosis pathways that are activated in response to different chemotherapeutic agents [21, 31], though this cytokine has also been reported to show anti-tumoral activities in certain circumstances [32]. In the current work, DOX-induced upregulation of P-gp and IL-6 will not only cause resistance to DOX but also many other anti-cancer drugs, a phenomenon termed multi-drug resistance (MDR) which is a major cause of chemotherapy failure. As opposed to DOX, Nano-DOX was neither subject to nor significantly induced P-gp and IL-6 (Figs. 4, 5, 6). It must also be mentioned that P-gp was not the only member in the ABC transporter family that was upregulated by DOX but not Nano-DOX. Another two important ABC transporters i.e. multidrug resistance-associated protein 1 (MRP1) and breast cancer resistance protein (BCRP) that are key mechanism in cancer MDR were also found upregulated by DOX but not Nano-DOX (data not shown). On the other hand, certain other cytokines e.g. IL-8 and GM-CSF saw a marked upregulation in 4T1 cells as well in response to DOX but not Nano-DOX (Additional file 1: Fig. S4). Through these factors, cancer cells can mobilize both intrinsic (autocrine) and external (paracrine) mechanisms to resist chemotherapy [33, 34]. We have further ongoing work exploring the mechanisms and therapeutic significance of these interesting phenomena. In summary, evasion of DOX-induced MDR is another advantage of Nano-DOX over DOX.

Immunosuppression, both at the systemic level and in local tissues, has been established as a hallmark of malignant tumors. Cancer-generated immunosuppression is mediated by various effector cells, among which MDSCs play a central role. MDSCs are a heterogeneous group of progenitor and immature myeloid cells which are blocked at early stages of maturation and differentiation. MDSCs have a potent immunosuppressive property which is best manifested as the capacity to inhibit T cell proliferation and activation. Many cancers can release haematopoietic cell growth factors such as G-CSF and GM-CSF which act on the bone marrow and strongly stimulate MDSCs expansion. For example, blood level of MDSCs in breast cancer patients can reach tenfold higher than normal [35]. MDSCs can infiltrate both tumor and normal tissues not only promoting tumor survival and progression but also thwarting various anti-cancer immunotherapies [36]. High MDSCs infiltration in tumor tissue has been associated with poor prognosis and therapy resistance in cancer patients [37]. Hence, therapeutic strategies to diminish MDSCs and/or modulate MDSCs functions have attracted great interest in basic and clinical cancer research and the therapeutic efficacy of certain anti-cancer drugs e.g. enthracyclines are associated with their ability to diminish MDSCs [38]. Indeed, here in this work, DOX exhibited significant toxicity to the ex vivo MDSCs (Fig. 3e) and diminished MDSCs both circulating in the blood and infiltrating tissues (Figs. 3a, 7a, b). On the other hand, Nano-DOX also reduced circulating MDSCs and tissue MDSCs infiltration (Figs. 3a, 7a, b). This may be due to a decrease of tumor-produced G-CSF secondary to suppressed tumor burden. Another cause of this effect may be Nano-DOX’s direct downregulation of 4T1 cell expression of G-CSF that fuels MDSCs expansion (Fig. 7c–e). Interestingly but paradoxically, serum G-CSF level showed a remarkable elevation in tumor-bearing animals in response to Nano-DOX and DOX (Fig. 7f). Similar observation has also been reported in clinical cancer treatment indicating the activation of an in vivo compensatory process in response to chemotherapy-induced myelosuppression [24, 25]. Concerning the cause of the elevated serum G-CSF level, there have been reports on lymphocytes as a compensatory source of G-CSF [39]. We have also detected increased G-CSF mRNA expression in leukocytes derived from the spleen but not blood of DOX-treated mice (data not shown) indicating lymphocytes in the spleen as a likely source of compensatory G-CSF. Diminished consumption of G-CSF due to reduced MDSCs burden might be another cause for the elevated serum G-CSF level after treatment [40]. It is noted that Nano-DOX’s MDSCs-diminishing activity was of a lesser strength than DOX (Figs. 3a, 7a, b). This is largely explained by the fact that Nano-DOX lacks DOX’s direct toxicity to immune cells including the MDSCs (Fig. 3e). Albeit hardly able to kill the MDSCs, Nano-DOX was found to reverse the phenotype of MDSCs induced by 4T1 cells. Nano-DOX caused a shift toward a more differentiated phenotype featuring upregulated Gr-1 and downregulated CD11b in both ex vivo bone marrow MDSCs and circulating MDSCs (Fig. 8). Naturally, alterations in the MDSCs’ cellular functions should underlie this phenotypic shift, which gives rise to an intriguing question: how will these altered MDSCs act back on the tumor cells? As a point of reference, we have both in previous [40] and current work (Fig. 10) demonstrated that Nano-DOX could reprogram the pro-tumor TAM (M2) into an anti-tumor phenotype (M1) through its action on the cancer cells. There have also been reports on MDSCs being re-polarized from a pro-tumor M2 phenotype to an anti-tumor M1 type [14, 41]. We have further study underway to explore using Nano-DOX to reprogram MDSCs. In comparison with Nano-DOX, DOX’s effects on MDSCs phenotype were overall of a similar pattern to Nano-DOX (Figs. 7, 8), indicating that Nano-DOX’s MDSCs-regulating activity may derive from DOX. However, DOX’s direct toxicity (Fig. 3e) may also affect MDSCs’ phenotype.

Like most other malignant solid tumors, TNBC also features a highly immunosuppressive local tumor microenvironment (TME) that is critically associated with the grim prognosis of the disease. TME consists some key types of immune cells including regulatory T cells (Tregs) and Mφ besides above-discussed MDSCs. Mφ generally fall into two activation states i.e. the anti-tumor, immunostimulatory M1 phenotype and the immunosuppressive, pro-tumor M2 phenotype. Mφ in the TME i.e. the tumor-associated Mφ (TAMs) are predominantly of the M2 phenotype that helps to suppress antitumor immunity and coordinate remodeling of the TME thus favoring tumor survival, growth, and progression [42, 43]. Of note, a subgroup of MDSCs will also differentiate into TAM of an M2 phenotype in the tumor microenvironment [44, 45]. The idea of reprograming the TAMs (M2) into the anti-tumor, immunostimulatory M1 phenotype has received great interest. On the other hand, the presence of tumor-infiltrating lymphocytes (TILs) particularly T cells, indicative of activation of anti-tumor immunity, in the tumor microenvironment have recently been associated with better prognosis of TNBC patients [46, 47] High extent of TILs are also predictive of improved response to chemotherapy and immunotherapy [48]. Thus, high hopes are pinned on increasing TILs in the tumor for improving patient outcome. Mφ reprograming and enhanced TILs presence in the tumor both require immunogenicity of the tumor and therapeutic strategies to stimulate tumor immunogenicity have been fervently explored. We previously have demonstrated that Nano-DOX could excite the immunogenicity of glioblastoma cells [13]. This action relies on Nano-DOX’s ability to induce autophagy in cancer cells and thereby stimulate emission of antigens and endogenous adjuvants i.e. the DAMPs [27]. There have been concerns that Nano-DOX’s ability to stimulate cancer cell immunogenicity might be cancer type-specific. In the present study, we showed pronounced DAMPs emission from the 4T1 cells, activation of Mφ phenotype from M2 to M1, DC activation and heightened presence and activation of TILs in the breast tumor in response to Nano-DOX (Figs. 9, 10, 11). These observations are compelling proof of Nano-DOX’s potency to stimulate the TNBC model’s immunogenicity despite that breast cancer generally is not a particularly immunogenic tumor. Taken together, the above results speak volumes for a third advantage of Nano-DOX which is its efficacy to reverse TNBC’s immunosuppression both at the systemic level and in the local TME.

Altogether, data obtained in this study demonstrate that Nano-DOX lacks the cytocidal activity of DOX but retains the cytostatic effect, leading to decreased tumor toxicity but increased host tolerance. This property confers upon Nano-DOX the advantage of selectively inhibiting the proliferating cancer cells with little adverse effect on the non-malignant cells, particularly the immune cells e.g. Mφ, DC and lymphocytes that are instrumental components of the anti-cancer immunity but are otherwise very sensitive to DOX’s toxicity. At the same time, Nano-DOX is a powerful immunoactive agent capable of reversing the cancer-induced immunosuppression both at the systemic level and in the TME by virtue of stimulating tumor immunogenicity and modulating cancer associated immune cells. Lastly but not least importantly, Nano-DOX circumvents the chemoresistance mediated by P-gp and IL-6. From a translational perspective, combination of Nano-DOX with immunotherapy may be a promising strategy for cancer treatment due to the nano-drug’s capacity to reverse tumor-induced immunosuppression. We have also in multiple tumor models observed that Nano-DOX upregulates PD-L1 in cancer cells and PD-1 in tumor associated immune cells and Nano-DOX has synergistic interactions with PD-L1/PD-1 inhibitors (unpublished data).

## Conclusions

In a nutshell, Nano-DOX is a cytostatic agent with good host tolerance which is capable of evading chemoresistance and reversing cancer-induced immunosuppression in TNBC. Nano-DOX also presents itself as an interesting example that a chemotherapeutic agent in nano-form may possess distinct biochemical properties from its free form, which can be exploited to join chemotherapy with immunotherapy for better treatment of cancer.

## Supplementary information

**Additional file 1: Figure S1.** In vivo fluorescent imaging of drug distribution. **Figure S2.** Fluorescent image of excised tumor xenografts showing Nano-DOX or DOX fluorescence in the tumors at 24 h after the last i.v. injection. **Figure S3.** Morphological observation of in-vitro 4T1 cells after 24-h treatment of Nano-DOX and DOX. **Figure S4.** Effects of Nano-DOX and DOX (2 and 4 μg/mL) on mRNA levels of IL-8 and GM-CSF in 4T1 cells. Duration of treatment was 24 h. **Figure S5.** Representative FACS histograms of HSP90 and CRT staining in 4T1 cells. **Figure S6.** Representative FACS histograms of CD40, CD80, CD83 and MHCII staining in activated DC. **Figure S7.** Representative FACS histograms of CFSE staining in activated lymphocytes. **Figure S8.** Representative FACS dot plots of CD69 staining in activated lymphocytes.

## Abbreviations

ATP: adenosine triphosphate
BM: bone marrow
b.w.: body weight
CFSE: carboxyfluorescein succinimidyl amino ester
CM: conditioned culture medium
CRT: calreticulin
DC: dendritic cells
DAMPs: damage-associated molecular patterns
DOX: doxorubicin
ER: estrogen receptor
ELISA: enzyme-linked immunosorbent assay
FACS: flow cytometry
G-CSF: granulocyte-colony stimulating factor
GM-CSF: granulocyte–macrophage colony stimulating factor
HER2: human epidermal growth factor receptor 2
H&E: hematoxylin and eosin
HMGB1: high mobility group protein B1
HSP90: heat shock protein 90
IHC: immumohistochemical
IF: immunofluorescence
ICD: immunogenic cell death
IL: interleukin
Mφ: macrophages
MDSCs: myeloid-derived suppressor cells
MDR: multidrug resistance
MFI: mean fluorescence intensity
Nd: nanodiamonds
ND: Nd-PG-DOX or Nano-DOX
ND-CM: conditioned medium by Nano-DOX (2 μg/mL)-treated 4T1
PR: progesterone receptor
P-gp: P-glycoprotein
PG: polyglycerol
PBS: phosphate buffered saline
PD-1: programmed cell death protein 1
PD-L1: programmed cell death-ligand 1
R123: rhodamin-123
TNBC: triple-negative breast cancer
TAMs: tumor-associated macrophages
TME: tumor microenvironment
Tregs: regulatory T cells
VRP: verapamil
