# Supplementary material for: Doxorubicin-polyglycerol-nanodiamond conjugate is a cytostatic agent that evades chemoresistance and reverses cancer-induced immunosuppression in triple-negative breast cancer
Source: J Nanobiotechnology. 2019 Oct 17;17:110. doi: 10.1186/s12951-019-0541-8 (PMC6798483; doi:10.1186/s12951-019-0541-8)
Supplement: Supplementary file 1 — Additional file 1: Figure S1. In vivo fluorescent imaging of drug distribution. Figure S2. Fluorescent image of excised tumor xenografts showing Nano-DOX or DOX fluorescence in the tumors at 24 h after the last i.v. injection. Figure S3. Morphological observation of in-vitro 4T1 cells after 24-h treatment of Nano-DOX and DOX. Figure S4. Effects of Nano-DOX and DOX (2 and 4 μg/mL) on mRNA levels of IL-8 and GM-CSF in 4T1 cells. Duration of treatment was 24 h. Figure S5. Representative FACS histograms of HSP90 and CRT staining in 4T1 cells. Figure S6. Representative FACS histograms of CD40, CD80, CD83 and MHCII staining in activated DC. Figure S7. Representative FACS histograms of CFSE staining in activated lymphocytes. Figure S8. Representative FACS dot plots of CD69 staining in activated lymphocytes. [file 12951_2019_541_MOESM1_ESM.doc]

**Additional Materials**


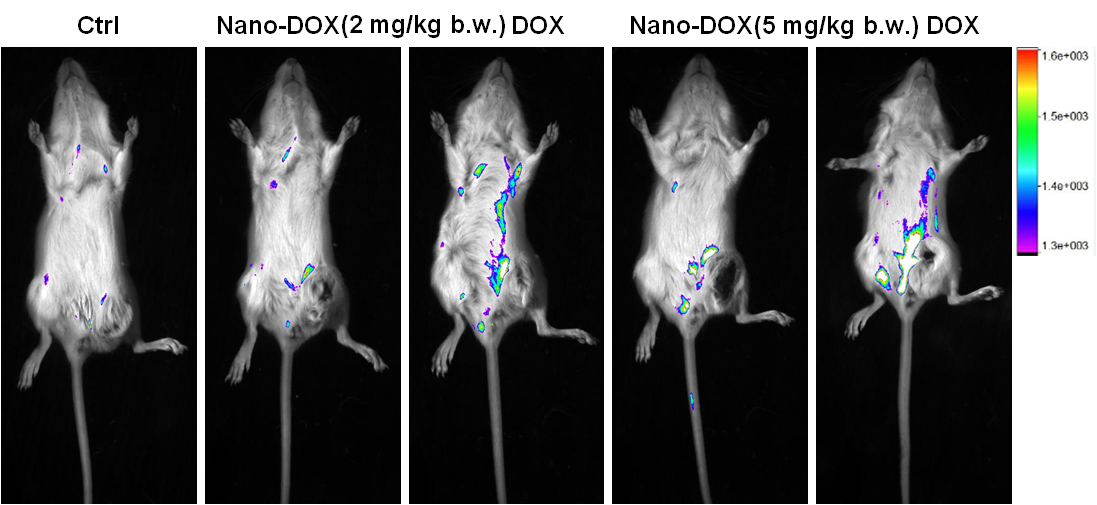


**Fig. S1.** Mice bearing orthotopic 4T1 xenografts were given introvenous injections of Nano-DOX or DOX (2 and 5 mg/kg b.w.) every 3 days for 3 weeks. Distribution of drug fluorescence was imaged 24 h after the last injection (representative graphs).


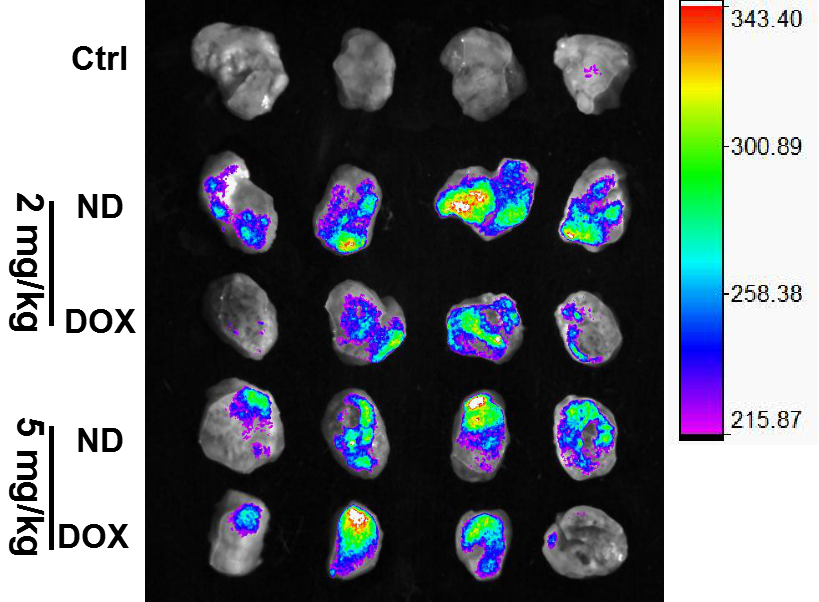


**Fig. S2.** Fluorescent image of excised tumor xenografts showing Nano-DOX or DOX fluorescence in the tumors at 24 h after the last i.v. injection. ND: Nano-DOX


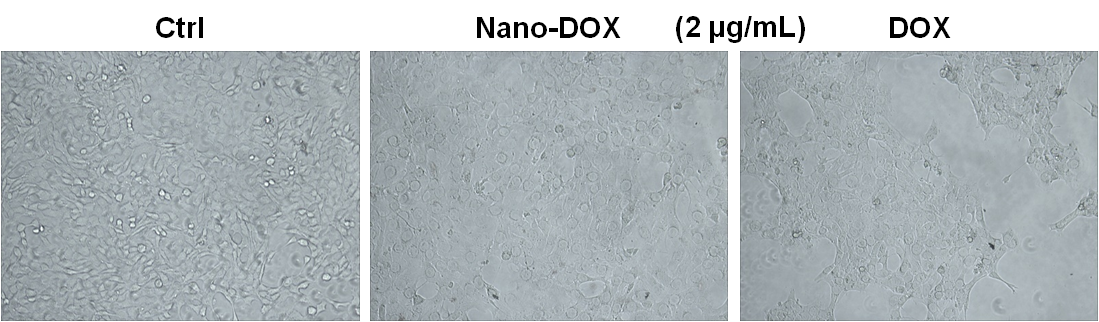


**Fig. S3.** Morphological observation of in-vitro 4T1 cells after 24-h treatment of Nano-DOX and DOX.


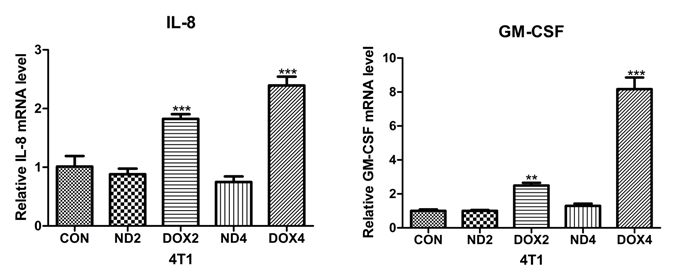


**Fig. S4.** Effects of Nano-DOX and DOX (2 and 4 μg/mL) on mRNA levels of IL-8 and GM-CSF in 4T1 cells. Duration of treatment was 24 h. Values were means ± SD (n = 3, **p < 0.01, ***p < 0.001).


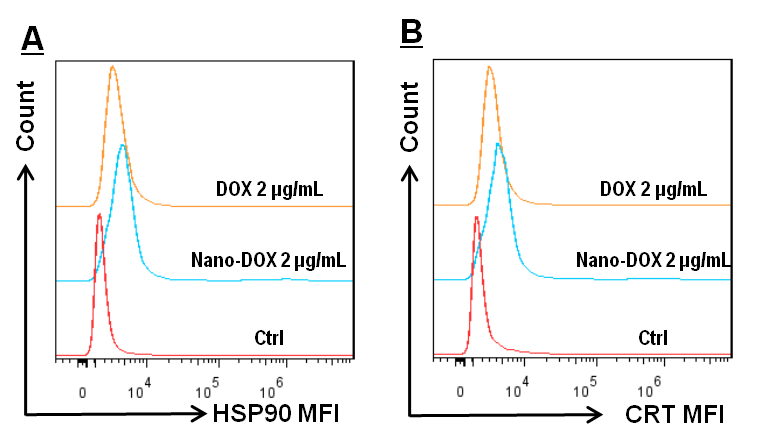


**Fig. S5.** Representative FACS histograms of HSP90 and CRT staining in 4T1 cells. See Fig. 9 in the manuscript for data. MFI: mean fluorescence intensity.


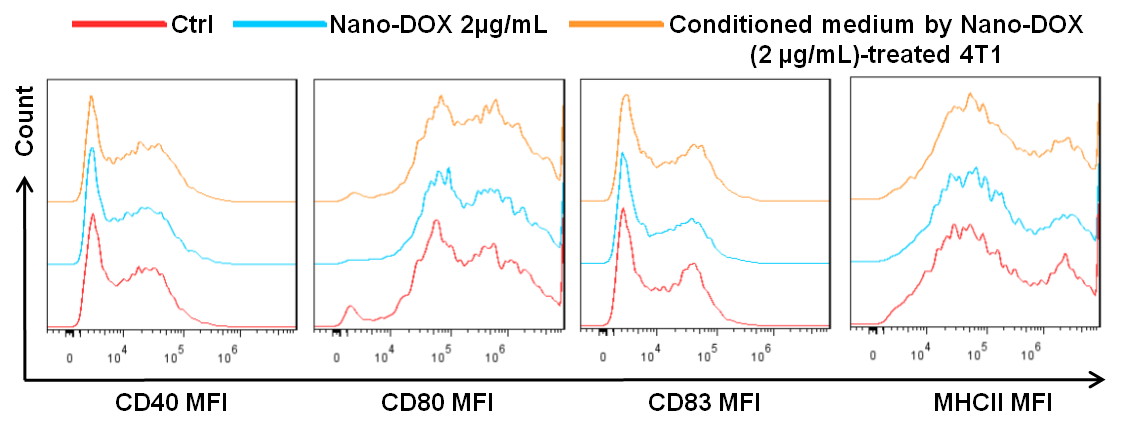


**Fig. S6.** Representative FACS histograms of CD40, CD80, CD83 and MHCII staining in activated DC. See Fig. 11 in the manuscript for data. MFI: mean fluorescence intensity.


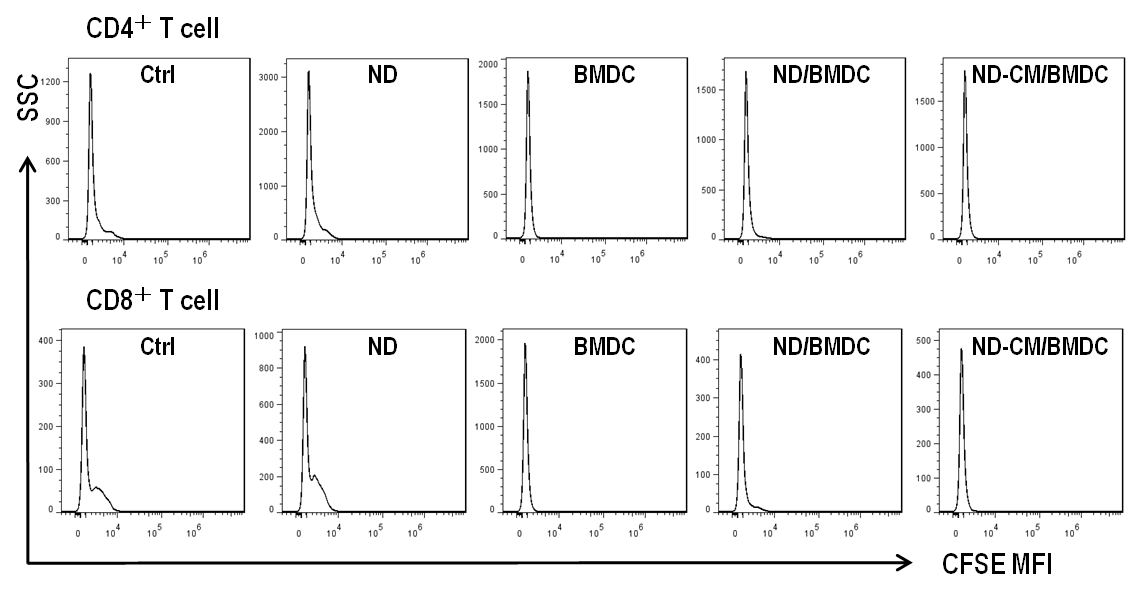


**Fig. S7.** Representative FACS histograms of CFSE staining in activated lymphocytes. See Fig. 11 in the manuscript for data. ND: Nano-DOX. ND-CM: Nano-DOX (2 μg/mL)-treated 4T1 cells. BMDC: bone marrow-derived dendritic cells. MFI: mean fluorescence intensity.


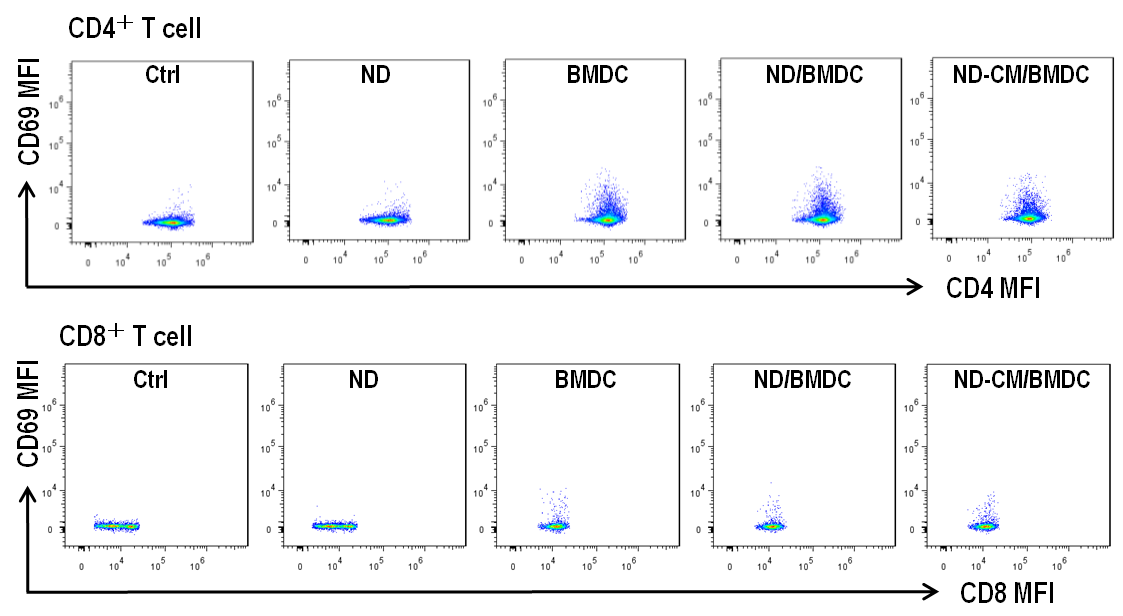


**Fig. S8.** Representative FACS dot plots of CD69 staining in activated lymphocytes. See Fig. 11 in the manuscript for data. ND: Nano-DOX. ND-CM: Nano-DOX (2 μg/mL)-treated 4T1 cells. BMDC: bone marrow-derived dendritic cells. MFI: mean fluorescence intensity.
